# Limited Scope for Group Coordination in Stylistic Variations of *Kolam* Art

**DOI:** 10.3389/fpsyg.2021.742577

**Published:** 2021-10-28

**Authors:** N.-Han Tran, Šimon Kucharský, Timothy M. Waring, Silke Atmaca, Bret A. Beheim

**Affiliations:** ^1^Department of Human Behavior, Ecology and Culture, Max Planck Institute for Evolutionary Anthropology, Leipzig, Germany; ^2^Department of Psychological Methods, University of Amsterdam, Amsterdam, Netherlands; ^3^Mitchell Center for Sustainability Solutions, School of Economics, University of Maine, Orono, ME, United States

**Keywords:** art, material culture, ethnic markers, coordination, cooperation, Bayesian inference

## Abstract

In large, complex societies, assorting with others with similar social norms or behaviors can facilitate successful coordination and cooperation. The ability to recognize others with shared norms or behaviors is thus assumed to be under selection. As a medium of communication, human art might reflect fitness-relevant information on shared norms and behaviors of other individuals thus facilitating successful coordination and cooperation. Distinctive styles or patterns of artistic design could signify migration history, different groups with a shared interaction history due to spatial proximity, as well as individual-level expertise and preferences. In addition, cultural boundaries may be even more pronounced in a highly diverse and socially stratified society. In the current study, we focus on a large corpus of an artistic tradition called *kolam* that is produced by women from Tamil Nadu in South India (*N* = 3, 139 *kolam* drawings from 192 women) to test whether stylistic variations in art can be mapped onto caste boundaries, migration and neighborhoods. Since the *kolam* art system with its sequential drawing decisions can be described by a Markov process, we characterize variation in styles of art due to different facets of an artist's identity and the group affiliations, via hierarchical Bayesian statistical models. Our results reveal that stylistic variations in *kolam* art only weakly map onto caste boundaries, neighborhoods, and regional origin. In fact, stylistic variations or patterns in art are dominated by artist-level variation and artist expertise. Our results illustrate that although art can be a medium of communication, it is not necessarily marked by group affiliation. Rather, artistic behavior in this context seems to be primarily a behavioral domain within which individuals carve out a unique niche for themselves to differentiate themselves from others. Our findings inform discussions on the evolutionary role of art for group coordination by encouraging researchers to use systematic methods to measure the mapping between specific objects or styles onto groups.

## 1. Introduction

Cooperation in humans requires groups of individuals to successfully coordinate and work together toward common or mutually beneficial goals. With the transition to agricultural societies, populations have become larger and more complex (Richerson and Boyd, 1999; Moffett, 2013). Thus, in these large, multi-ethnic societies assorting with others with similar social norms or behaviors can facilitate successful coordination and cooperation (Hamilton, 1964a,b; Axelrod and Hamilton, 1981). In evolutionary biology, an abundance of research has focused on mechanisms that allow individuals to interact preferentially with each other, showing that cooperation can evolve and stabilize when individuals preferentially interact with close kin or have a recognition mechanism to interact with other individuals with cooperative traits or shared norms (McElreath et al., 2003; Gintis, 2014). Thus, the ability to recognize others with similar social norms or behaviors is presumably under selection (Riolo et al., 2001; Jansen and Van Baalen, 2006).

As a medium of communication, human art might reflect fitness-relevant information on shared norms and behaviors of other individuals thus facilitating successful coordination and cooperation. Distinctive styles or patterns of artistic design could signify migration history, different groups with a shared interaction history (e.g., kin, neighbors, or members of the same caste) or individual-level variation and expertise (i.e., duration of practice). To investigate the long standing and growing interest in quantitative detection of ethnic markers across disciplines, we present an Bayesian analysis of a large corpus of material art created by women from Tamil Nadu in South India, called *kolam* drawings. Using this corpus of *kolam* art, we test whether stylistic variations in art can be mapped onto caste boundaries, migration and neighborhoods, and how much variation in styles can actually be accounted for by these factors. Specifically, we illustrate how we can exploit the Markovian nature of the art system to our advantage to build a hierarchical model that is able to describe and partition the variation in the complex, sequential drawing compositions in order to better understand the role of art for social coordination.

### 1.1. Theoretical Background

In ethnic marker theory, it is assumed that human groups have developed distinct and overt ethnic markers or tags to signal group membership to preserve cultural boundaries (Barth, 1969; McElreath et al., 2003; Moffett, 2013). The use of ethnic markers can help solve coordination and collective action problems because they communicate interaction norms. Ethnic markers can be manifested in various forms from sartorial cues, dialectic variations, special adornments to distinct styles in production (Wobst, 1977). Thus, mastering different styles of weaving (Tehrani and Collard, 2009), arrow head production (Wiessner, 1983) or pottery (Bowser, 2000) could be fundamental for social interaction because of the fitness-relevant information about with whom, when, and how to interact. For instance, in the Ecuadorian Amazon, women's pottery style reflects their social identity and group membership as part of their political strategies (Bowser and Patton, 2008). Learning to identify, create, and modify stylistic symbols in pottery play a fundamental role in women's lives and social standing with Achuar and Quichua society.

While theoretical models and experiments in the laboratory demonstrate the mapping between group membership and objects for the purpose of social coordination (McElreath et al., 2003; McElreath and Boyd, 2007; Efferson et al., 2008), evidence from the field has been more ambiguous about the link between objects or styles and groups for social coordination (Hodder, 1977; Wiessner, 1984; Moya and Boyd, 2016). Thus, Bell (2020) have pointed out the need for a systematic method that is able to measure the mapping between a specific object or style onto groups because such a statistical measure of an object's role in social coordination has been largely elusive. In fact, Bell and Paegle (2021) presented a three-step ethnographic field method, consisting of scans, surveys, and classification tasks, to assess the role of motifs for social coordination. Specifically, the triad classification task was demonstrated to systematically measure whether motifs have information content as a result of population-specific socialization. This approach from Bell and Paegle (2021) is well-suited for systems with a finite set of motifs. A complementary approach to the field methods from Bell and Paegle (2021)—however, still elusive—would be a quantitative approach on the corpus of artistic productions that is able to identify styles or patterns that are salient *de novo*, not constrained by functional requirements and associated with groups or individuals.

### 1.2. *Kolam* Art

*Kolam* designs are ritual patterns that Tamil women draw with rice powder or chalk on the threshold of their houses in South India (Durai, 1929; Layard, 1937). Each morning before sunrise, women typically clean the thresholds of their homes and then start to draw *kolam* loop patterns by initializing a grid of dots (Laine, 2013). Subsequently, continuous lines are drawn around the dots to form intricate loop patterns.

While Hindu women draw threshold designs throughout India (e.g., rangoli in Uttar Pradesh or mandana in Rajasthan; Kilambi, 1985; Saksena, 1985), *kolam* designs are specific to Tamil Nadu (Layard, 1937; Laine, 2013). As a symbol of generosity, *kolam* drawings are a ritual offering to animals “to feed a thousand souls” (Nagarajan, 2018, p. 56, 243–255). Most importantly, the *kolam* communicates the state of the artist and its household, and marks important events within the household as well as in the village (Nagarajan, 2018, p. 37, 52–55, 75–81, 267). On the one hand, *kolam* designs communicate to neighbors and visitors that the household is healthy with sufficient food and able to host guests and be hospitable. Thus, especially large and complex *kolam* loop patterns are drawn on auspicious events, such as weddings or births. In contrast, the absence of *kolam* drawings typically indicates inauspicious events, such as death or menstruation, and thus signifies the inability to host visitors. On the other hand, *kolam* designs are further understood to convey information on the artist's personality (e.g., womanliness, traditionalness, and patience) and their competency to run a household and become a good wife and mother.

*Kolam*-making is not formally taught in school or training institutions, but knowledge is mostly transmitted from (grand-)mothers to (grand-)daughters and accumulated through practice and exposure over time (Nagarajan, 2018, p. 67–69). Since *kolam*-making and mastery are considered necessary for the transition into womanhood, women start to learn and practice *kolam*-making from an early age of about 6 years (Nagarajan, 2007, p. 8, 12, 156). Diligent practice in private notebooks would be required until a *kolam* design is finally showcased on the threshold of the household. It takes approximately 6 years to master the ability to draw a beautiful and complex *kolam* with continuous lines that do not intersect with the dots, uniform line widths, and invisible starts and stops of loops (Nagarajan, 2007, p. 128, 156). While hand-drawn *kolam* designs are never for sale (Nagarajan, 2018, p. 36), women can buy design books that display a variety of printed example *kolam* designs. Since the *kolam* traditions and the designs are considered community knowledge (Nagarajan, 2018, p. 69), women would often come together to share their designs with each other. In anticipation of an auspicious event, women in the household would share their ideas and carefully plan the especially complex *kolam* pattern of that day. At the same time, women would compete with each other to draw the most innovative, dense, and geometrically complex pattern during festivals or contests (Nagarajan, 2018, p. 179–203).

### 1.3. Current Study

In the current study, we focus on Tamil *kolam* art to demonstrate a novel approach to quantify covariation of artistic styles along cultural boundaries. The *kolam* system is well-suited to study social coordination because *kolam* art is observable (e.g., women display *kolam* drawings on their thresholds), recognizable (e.g., *kolam* art is specific to Tamil Nadu and has a specific grammar) and plays an important role in Tamil culture with an abundance of artists (and their multitude of group identities) learning and producing *kolam* drawings from a young age. Social learning and the accumulation of *kolam* knowledge across generations could have led to the covariation of styles or patterns in *kolam* art along certain cultural boundaries. Since *kolam* drawings arise in a highly stratified, caste-based society, the question arises whether different styles in *kolam* art may communicate group membership for social coordination.

*Kolam* designs actively broadcast information about the household to neighbors and visitors. For instance, a consistent absence of *kolam* patterns on the threshold indicates that the household is not Hindu (Nagarajan, 2018, p. 75). Furthermore, caste distinctions are reported among *kolam* designs with, for example, certain styles dominating between different subgroups of the Brahmin caste (Saroja, 1988; Nagarajan, 2018). According to Nagarajan (2018, p. 149), “regional variations are recognized and regularly discussed among women”. Thus as predicted by ethnic marker theory (McElreath et al., 2003), the mapping between *kolam* styles and group identities should become more salient at these cultural boundaries. However, a concrete quantification whether and to what extent differences in *kolam* styles are associated with caste, region, or other variables is still elusive (Nagarajan, 2018, p. 273) and requires a systematic investigation.

*Kolam* drawings cannot only be mapped onto a small identifiable set of gestures^1^ “with systematic procedures and techniques” to create them (Ascher, 2002, p. 5), but their beauty is also characterized by continuous lines and loops with smooth transitions between gestures (Nagarajan, 2018, p. 128, 156). Thus, this naturalistic art system can be described by a state-based Markov process due to its series of sequential and dependent decisions. This Markovian nature of the art system allows us to build hierarchical statistical models that are able to account for variation in styles in art (i.e., the complex composition of gesture sequences that result in motifs, patterns, or styles in artistic design) due to different facets of an artist's social identity or their group membership, thus elucidating the role of the complex composition of gestures in social coordination. In the current study, we describe (1) the general styles or patterns of *kolam* designs, and further investigate (2) whether stylistic variations in artistic design can be linked to caste boundaries, migration and groups of individuals with a shared interaction history due to spatial proximity (neighborhood), and (3) how much of the stylistic variations in artistic design can be accounted for by artist-level variation and group affiliations relative to each other.

## 2. Methods

### 2.1. Data

We used a data set of 3, 139 *kolam* drawings (on average 16 *kolam* per woman) from 192 artists (age: mean = 31.88 years, sd = 10.08 years, range = 15−60 years; married: 73%, non-native ≈18%) that were collected in Kodaikanal, Tamil Nadu in South India in 2009 by TMW and local research assistants. A survey was conducted on artists' *kolam* drawing abilities and behavior and other demographic information, such as their age, marital status, caste membership, number of children, the years of *kolam* practice, and their migration background (i.e., nativity). Artists in our data set self-identify with a total of 19 different caste categories. These caste categories are associated with varying privileges and include local and migrant caste groupings. Additionally, the spatial proximity of each artist to each other was measured.

**Figure 1:**
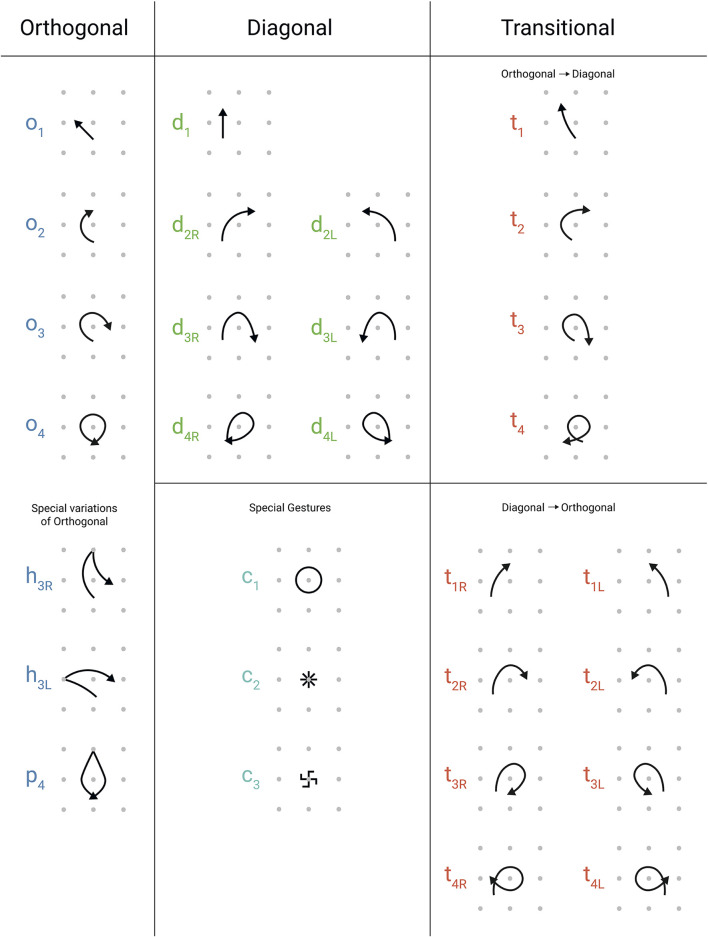
The Lexicon of *Kolam* Gestures. This figure illustrates the gestures and the corresponding code to encode *kolam* drawings. Taken and adapted with permission from (Waring, 2012b).

A *kolam* drawing can be constituted of one or more closed loops. Each loop can be decomposed into a sequence of gestures using a lexicon of gestures (Waring, 2012b). A complete description of the lexicon can be found in Figure 1. The lexicon of gestures contains 29 gestures, denoting the geometric space of the gestures as well as the chirality of gestures with distinct left and right versions since rotations of these gestures in space cannot yield their exact mirror image (see Figure 1, lower row right). The gestures that constitute a loop and the *kolam* drawings can be categorized into three different geometric spaces with distinct characteristics: orthogonal, diagonal, and transitional (each set of gestures represented by O, D, T, respectively). Additionally, there are three special single gestures that serve as decoration and are not part of a loop. We transcribed the *kolam* data using the lexicon.

#### 2.1.1. Geometric Spaces in *Kolam* Art

**Figure 2:**
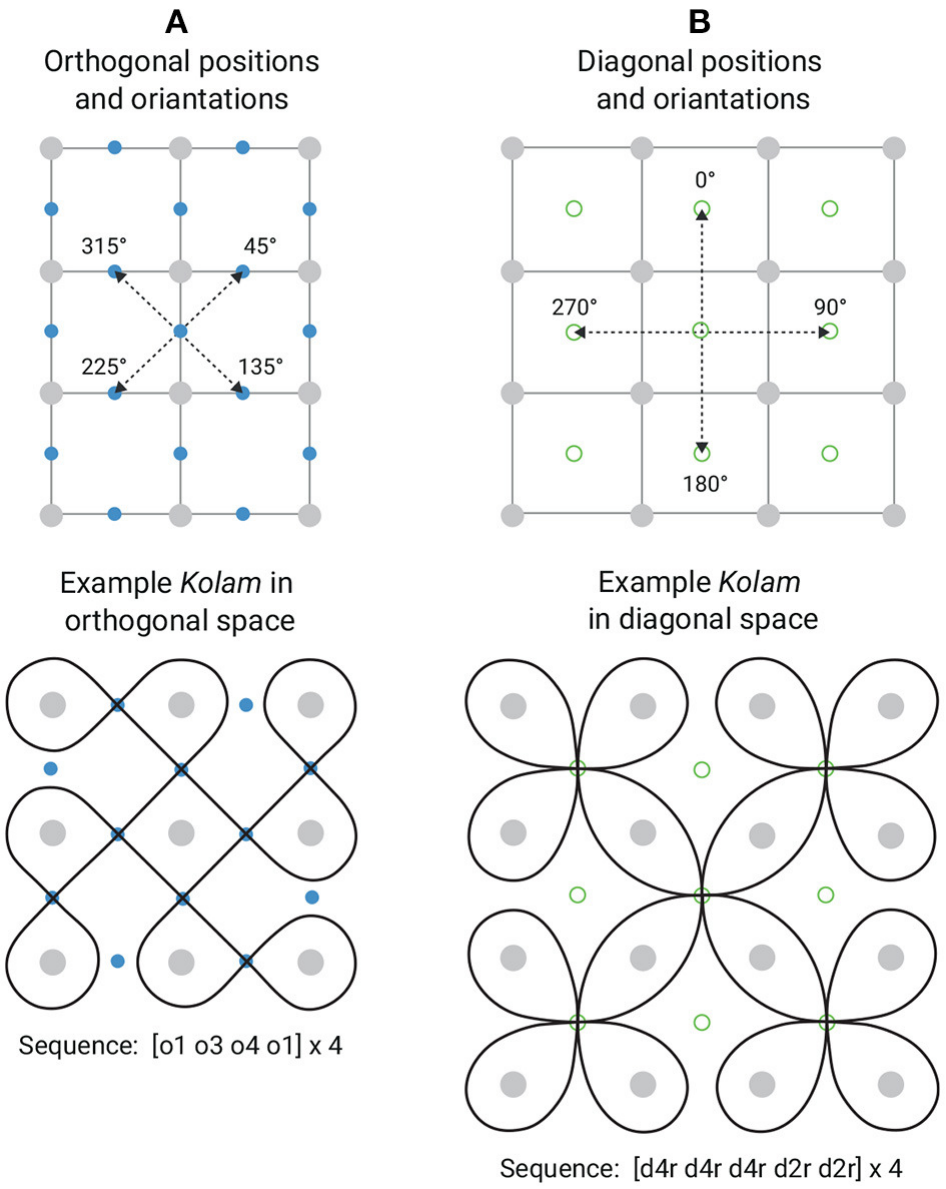
Explanation of the geometric spaces in *kolam* art. Taken and adapted with permission from Waring (2012b). Panel **(A)** shows the orthogonal geometric space with an example *kolam* drawing and Panel **(B)** shows the diagonal space with an example *kolam* drawing. In Panel **(A)**, the dashed arrows illustrate the possible orthogonal starting and ending positions of gestures (blue dots) and loops as well as the orthogonal orientations of gestures: 45°, 135°, 225°, 315° angles. In **(B)**, the dashed arrows illustrate the possible diagonal starting and ending positions of gestures (green circles) and loops as well as the diagonal orientations of gestures: 0°, 90°, 180°, 270° angles. Sequence: The sequence of gestures that the specific *kolam* designs can be decomposed into (see lexicon in Figure 1).

*Kolam* patterns have a grammar that contains many mathematical principles (Siromoney et al., 1974; Ascher, 2002; Waring, 2012b). Specifically, we refer to three geometric spaces that determine the starting and ending positions of a loop as well as the orientation of the loops: orthogonal, diagonal and transitional space. Figure 2 illustrates orthogonal and diagonal spaces with example *kolam* drawings each. While orthogonal loops (i.e., loops in orthogonal space) start and end between two neighboring dots, diagonal loops (i.e., loops in diagonal space) start and end in the center of four dots. Furthermore, orthogonal gestures are oriented toward 45°, 135°, 225°, or 315° angles, and diagonal are oriented toward 0°, 90°, 180°, 270° angles. Gestures that start in orthogonal space, end in orthogonal spaces. Gestures that start in diagonal space, end in diagonal space. Thus, orthogonal and diagonal gestures are disjoint, but can be connected with each other using transitional gestures. Transitional gestures can either start in diagonal space and end in orthogonal space or they start in orthogonal space and end in diagonal space. Since orthogonal and diagonal gestures have distinct starting and ending positions and orientations, and transitional gestures share orthogonal and diagonal positions and orientations, switching between these different geometric spaces requires practice in order to still maintain smooth transitions, continuous loop closures, and uniform line-widths. Thus, *kolam* designs in only one geometric space tend to be easier to create, especially if the *kolam* design only consists of orthogonal gestures.

#### 2.1.2. *Kolam* Art as a Markov System

Since each *kolam* loop pattern^2^ can be decomposed into a sequence of gestures and artists strive to form uniform and smooth loops, the system can be described by a state-based Markov process, whereby the conditional probability distribution for the system at the next step depends only on the current state of the system, and not on the state of the system at a previous step (Gagniuc, 2017).

**Figure 3:**
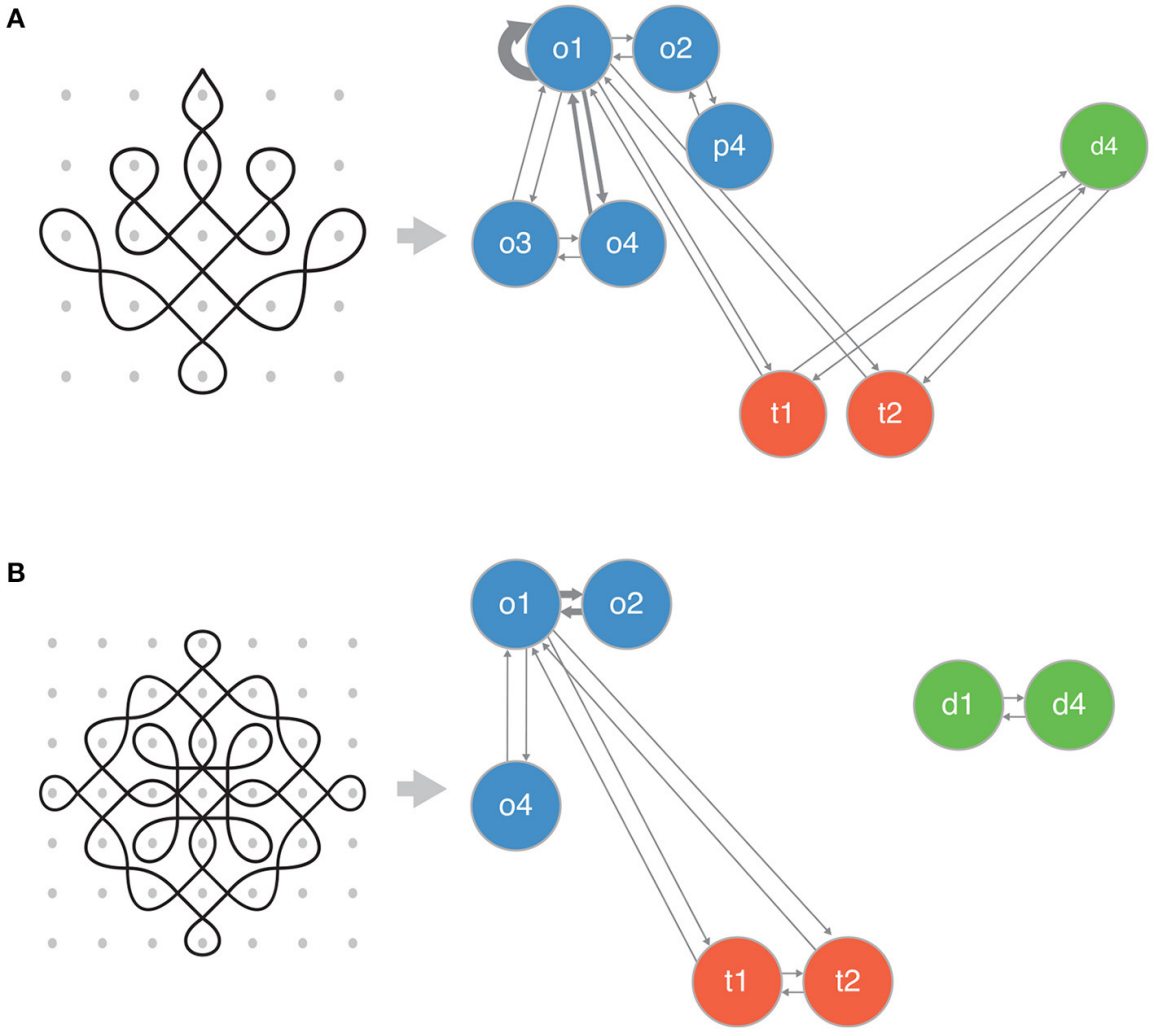
Example of a transition (count) networks for *kolam* drawings. Each row **(A,B)** corresponds to an example *kolam* drawing from an artist. The edge width represents the count, whereby an increased width implies a higher transition count. The three colors represent the different geometric spaces: orthogonal (blue), diagonal (green), and transitional (orange). Each node represents a gesture used by the artist from the lexicon of gestures (see Figure 1).

Each gesture within a geometric space can be considered a state and thus, each loop and *kolam* drawing can be described by a probabilistic state transition matrix *m*×*m* where the row vector *m*×1 describes the state (i.e., gesture) and column vector the transition to the next state (i.e., gesture). Every gesture is accessible from itself. Furthermore, the gestures in this Markov system can be partitioned into communicating classes such that gestures of the same geometric space communicate with each other (accounting for chirality), and gestures of orthogonal and diagonal space are only connected via gestures of transitional space (see section 1.1 for more details). Figure 3 illustrates the transition count matrix for *kolam* drawings from two example artists.

We computed the transition counts for each loop of a *kolam* drawing across all the *kolam* drawings of an artist. The three special, single gestures were not considered because they are not part of a loop. Since each of the sequences of gestures represents a loop and the artists' starting location of a loop or *kolam* drawing is unknown, we further counted the transition from the last gesture in the sequence to the first gesture in the sequence. These transition counts not only reflect the distribution of gestures for each artist, but further artists' preferences or biases toward specific patterns or motifs (i.e., specific sequence of gestures). We first computed an aggregated transition count matrix *y*_*i*_ for each artist *i* of size 14 × 14. In order to fit our full model, we further computed four transition matrices *y*—one for each of the different geometric spaces and one for the transitions between geometric spaces: transitions between geometric spaces of size 3 × 3, transitions within orthogonal space of size 6 × 6, transitions within diagonal space of size 4 × 4 and transitions within transitional space of size 4 × 4. These transition matrices *y*_*ijk*_ represent the count of transitions from state (i.e., gesture) *j* to state (i.e., gesture) *k* for artist *i*. Thus, distinct patterns of *kolam* drawings can arise from different transition probabilities *within* geometric spaces and *between* geometric spaces—if any transition between geometric spaces occurs at all.

### 2.2. Statistical Analyses

To investigate the variation in *kolam* patterns and motifs due to individual variation, social stratification reflected by caste membership, nativity or neighborhood (i.e., place of residence), and expertise measured by the years of practice, we fitted a total of seven Bayesian statistical models. Here, we will only focus on the full model, but the interested reader can see the details of all the models in the Supplementary Material. For each artist, we modeled four transition matrices. The statistical model reflects the process of how the *kolam* drawings arise; the probability of the next state (gesture in a geometric space) is conditional on the current state. In the current model, the conditional probability of the next state given another state is factored into two components. One component encodes the probability of transitioning between geometric spaces; the other encodes the probability of transitioning between gestures within a geometric space. For instance, given that the current state is the gesture *o1*, then the probability that the next gesture will be *o2* is comprised of the probability of staying in the current orthogonal geometric space *O* times the conditional probability of choosing gesture *o2* given being currently in gesture *o1*: *P*(*o*2|*o*1) = *P*(*O*|*O*) × *P*(*o*2|*o*1, *O*|*O*).

Each row of each transition matrix is probabilistic and modeled on the logit scale using the softmax link (i.e., multinomial logistic regression). Caste was modeled as a varying effect with 19 categories to estimate individual offsets for each caste category. Since there are multiple *kolam* drawings for each individual, caste group and neighborhood, information across individuals, castes and neighborhoods was partially pooled using hierarchical modeling to account for imbalances in sampling and yield more reliable and precise estimates (Efron and Morris, 1977). The duration of practice was standardized to be centered on zero with a standard deviation of one. The native place was a binary indicator predictor variable (0 = native, 1 = non-native). A geodesic distance matrix was computed between the GPS coordinates and subsequently hierarchically clustered with a distance threshold of 500m, resulting in 8 neighborhood clusters. The neighborhood clusters were modeled as a varying effect with 8 categories to estimate individual offsets for each neighborhood.

The models can be parameterized differently by including or excluding predictors as well as setting equality constraints for various parameters between rows of the transition matrices. We used leave-one-out cross-validation (Vehtari et al., 2017) and computed Pseudo-Bayesian model averaging^3^ and stacking weights (Yao et al., 2018) using the log-likelihood evaluated at the posterior simulations and the R package loo (Vehtari et al., 2020) to estimate and compare out-of-sample prediction accuracy of our fitted models.

The statistical models were implemented in the probabilistic programming language Stan (v2.18) (Carpenter et al., 2017), using 4,000 samples in four independent chains. All $\hat{R}$ values were less than 1.01, and visual inspection of trace plots, rank histograms and pairs plots indicated convergence of all models (see Supplementary Section 4.1 for more details). A principled and robust Bayesian workflow with an iterative process of model building, inference, model checking and evaluation, and model expansion was used (Talts et al., 2018; Gabry et al., 2019). Prior predictive simulations were used to determine weakly informative priors for the parameters. Thus, there were no indication of convergence issues and the models were optimally calibrated. We present a complete description of the statistical models and the priors in the Supplementary Section 2.7. Data and analyses can be found at http://github.com/nhtran93/kolam_coordination.

### 2.3. Intraclass Correlation (ICC)

The intraclass-correlation coefficient (ICC) can be calculated to determine the proportion of the total variance explained by random and fixed effects (Nakagawa et al., 2017). We calculated a modification of the ICC using variance decomposition of the model predictions^4^. We drew predictions of the transition probabilities on the logit scale using the posterior samples for each of our fixed (i.e., migration, duration of practice) and random (i.e., neighborhood, caste, individual variation) terms separately, using the estimates from the full model. Thus, for each fixed or random term, we obtain the transition probabilities (on the logit scale) implied by that term in isolation for each individual. The ICC is then the ratio between the variance of the predictions across individuals from a single term divided by the sum of the variances of predictions across individuals for all terms ($ICC_{i}=\frac{Var\left({\text{predictions}}_{i}\right)}{\sum Var\left({\text{predictions}}_{j}\right)}$). This calculation was done per each MCMC iteration and each cell of the transition matrix separately. The final estimates are based on the average over the MCMC iterations and all cells in the transition matrix^5^. The value of ICC corresponds to the proportion of total variance of the model's transition probabilities on the logit scale that is accounted for by a particular term in the model. By construction, the coefficient cannot be smaller than 0 and the sum of all ICCs is equal to 1. Thus, the ICC quantifies the proportion of the total variance explained and is suitable to compare relative strength of the model terms. However, the absolute strength of each predictor is dependent on all other terms in the model.

## 3. Results

According to Pseudo-Bayesian model averaging and stacking weights in leave-one out cross-validation, our full model has the best predictive performance (see Supplementary Section 3 for more details).

On the population-level, our results illustrate that although artists are unconstrained in their patterns or stylistic variation in *kolam* drawings and they can freely transition back and forth between geometric spaces and gestures, artists have evident preferences and biases toward certain gestures and geometric spaces. *Kolam* patterns that arise in orthogonal geometric space are predicted to stay in orthogonal geometric space with a probability of 0.99 and transitioning to a different geometric space from orthogonal space to access a greater diversity of gestures hardly occurs with a probability of 0.01 (see Table 1). As seen in Table 1, if an artist draws a *kolam* artwork in diagonal space, they are predicted to equally likely switch to transitional space (probability of 0.51) or remain in the current diagonal space (probability of 0.49), while artists that draw a *kolam* artwork in transitional space are predicted to remain in the current space with a probability of 0.30 and switch to orthogonal or diagonal space with a probability of 0.51 and 0.19, respectively. Therefore, when an artist draws kolam patterns in orthogonal space, they are unlikely to transition between different geometric spaces and only draw patterns with different gestures within the orthogonal space. However, if artists draw kolam patterns in diagonal or transitional space, they tend to use a diverse set of gestures that span across multiple different geometric spaces. For a more in-depth analysis of the population-level tendencies in gesture compositions that result in specific gesture equilibria and styles in artistic design, please consult the Supplementary Section 4.2.

**Table 1:** Population-level estimated posterior transition matrix across geometric spaces.

	**Orthogonal**	**Transitional**	**Diagonal**
Orthogonal	0.99	0.01	0.00
Transitional	0.51	0.30	0.19
Diagonal	0.00	0.51	0.49

**Figure 4:**
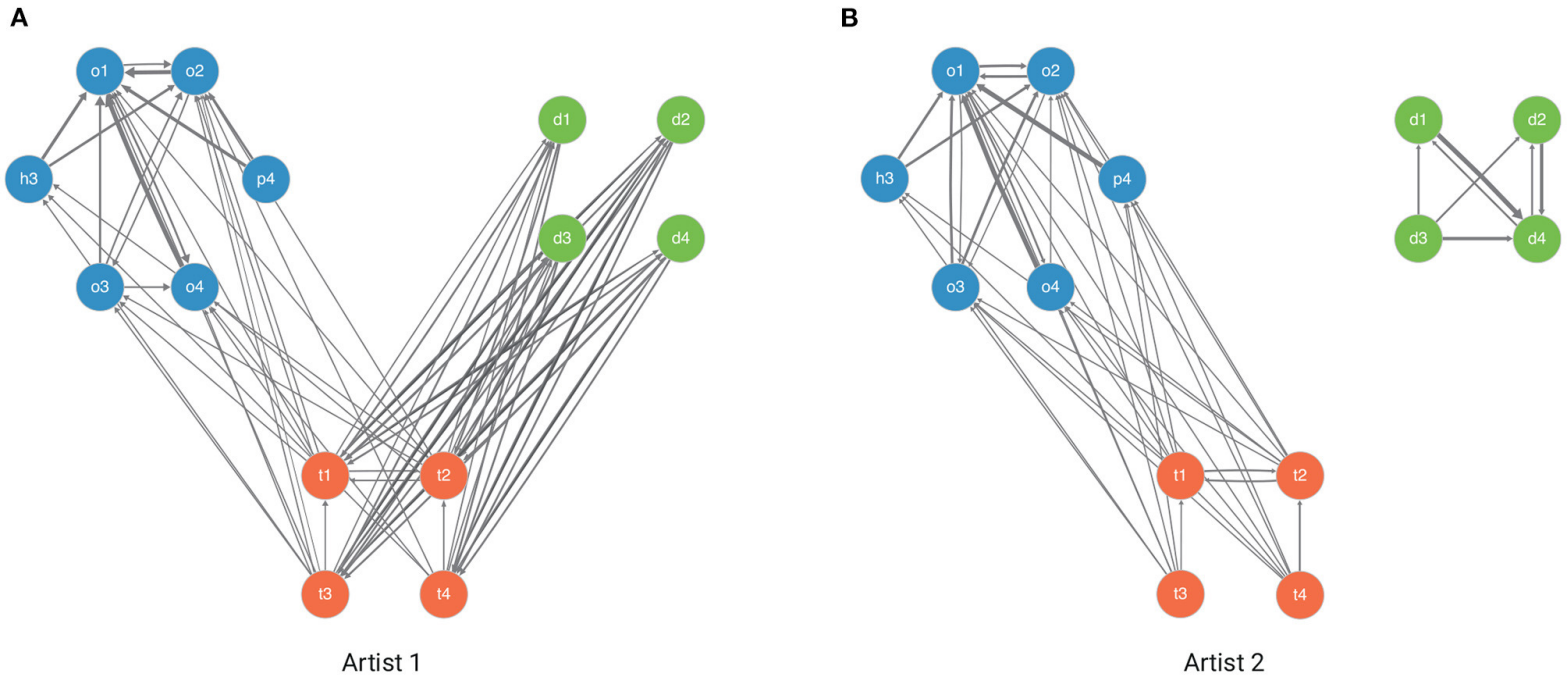
Estimated individual-level transitions between orthogonal, diagonal and transitional gestures for two example artists. Each panel represents an example artist (**A**: artist 1, **B**: artist 2) with her corresponding estimated transition probabilities between gestures. The width of the edges reflects the probability of transition, whereby a wider or bolder edge implies an increased probability of transition. Self-loops are not displayed.

Figure 4 shows the estimated individual-level transitions between orthogonal, diagonal, and transitional gestures from two artists and an example *kolam* artwork each from their repertoire. As seen in Figure 4, artists' styles in drawing *kolam* artwork can be very divergent between artists. While the artist corresponding to the estimated transitions and example *kolam* artwork in Figure 4A would draw *kolam* artworks that arise and remained in orthogonal space with a probability of 0.846 at equilibrium, she would only occupy diagonal (0.041) and transitional (0.113) spaces at equilibrium. The artists corresponding to Figure 4B would create *kolam* artworks that occupy orthogonal (0.53), diagonal (0.35), and transitional (0.12) spaces at equilibrium. Furthermore, in contrast to the artist corresponding to Figure 4A, the artist corresponding to Figure 4B draws diagonal gestures in isolation without connecting their gestures to gestures from other geometric spaces. In fact, the *kolam* designs displayed in Figure 3 correspond to the artists in Figures 4A,B, respectively. Thus, the vastly different transition probabilities between gestures among artists can thus give rise to diverse patterns or motifs in *kolam* artwork.

**Figure 5:**
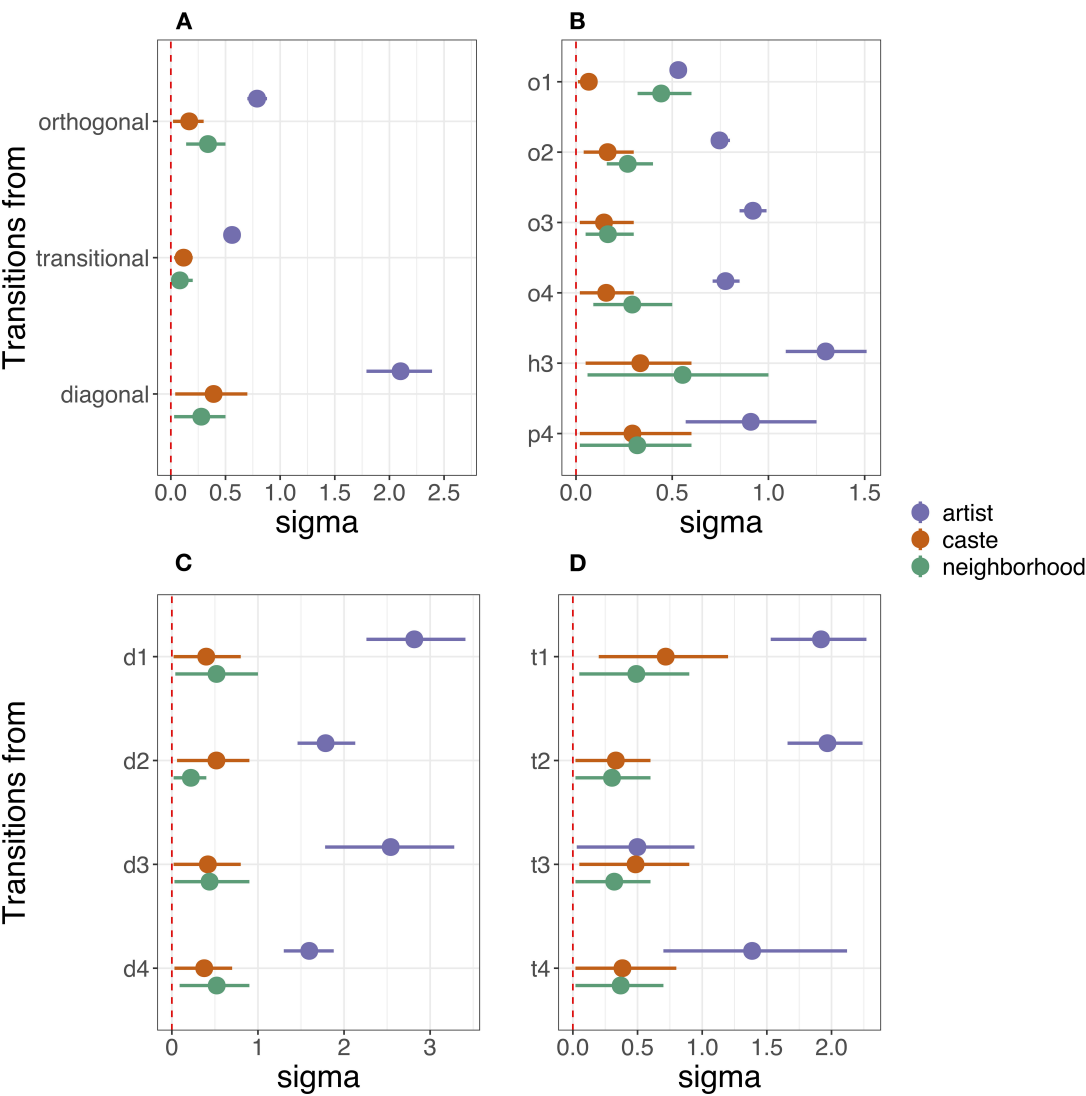
Posterior Coefficient Plots displayed as the 90% Highest Posterior Density Interval (HPDI) of the posteriors of sigma estimates (i.e., standard deviation of the random effects) associated with variation due to artists, caste, and neighborhood. Transitions *from* geometric spaces **(A)** and *from* individual gestures **(B–D)** are shown on the *y*-axis.

Even though the estimated population-level transition matrices reveal a tendency in how kolam patterns arise, our results show that there is substantial variation in kolam drawing styles and patterns between artists. Whether styles in kolam drawings comprise of one or more transitions between geometric spaces or only arise and remain in the same geometric space, our results reveal that most of the variation in styles or patterns of kolam artwork is driven by individual differences and their expertise (see Figure 5 and Table 2). Figure 5 shows the size of the variance estimates (i.e., random effects) in transitioning to the next gesture from a given geometric space (Figure 5A) or gesture (Figures 5B–D). The variance parameters for the transition probabilities in *kolam* artwork are dominated by artist-level variation, whereby the artist-level variation has consistently the largest estimate on all four transition matrices. As illustrated in Figure 5, caste membership and neighborhood have smaller estimates on the four transition matrices. The duration of practice shows large estimates on the gesture transitions within diagonal and transitional space and moderately large estimates in transitions between diagonal and transitional space (see Supplementary Section 4.3). Former migration history further only shows small estimates on the transition between geometric spaces, while the estimates for within orthogonal and transitional space transitions are moderately large (see Supplementary Section 4.4).

**Table 2:** Explained variation (intraclass correlation).

	**Individual**	**Expertise**	**Nativity**	**Caste**	**Residence**
Across geometric spaces	0.63	0.25	0.06	0.03	0.04
Orthogonal	0.62	0.20	0.05	0.03	0.10
Diagonal	0.83	0.07	0.02	0.05	0.03
Transitional	0.60	0.16	0.06	0.12	0.06

According to the computed ICCs, *kolam* drawing styles with one or more transitions between different geometric spaces largely map onto individual variation (63%), so that the variance of predictions from a model with just individual artists' intercepts make up 63% of the total variance; the expertise of the artist accounts for additional 25% of the total prediction variance (see Table 2). In other words, on average 63% of the variation in the predicted transition patterns between geometric spaces can be attributed to the variation between distinct artists unexplained by other predictors, and a further 25% can be attributed to their expertise, measured as the duration of practice. Individual variation (62%) paired with artist's expertise (20%) further primarily accounts for styles of *kolam* artwork that arise and remain in orthogonal space (see Table 2). Caste membership can account for 12% of the variation in transitions between transitional gestures in *kolam* artwork, but can only account for very little variation in the rest of the estimated transition probabilities (see Table 2). Similarly, previous migration history or the neighborhood of the artist can explain 10% of the variation in transitions between orthogonal gestures, but only little in the rest of the estimated transition probabilities in *kolam* artwork (see Table 2).

## 4. Discussion

Our statistical model revealed that styles or patterns in *kolam* artwork can only be very weakly mapped onto group affiliations, such as caste boundaries, neighborhoods, or previous migration. Styles or patterns in *kolam* artwork show group-level variation; however, the group-level variation is limited. Hence, it is unlikely that styles or patterns in this artistic tradition operate as ethnic markers. Albeit artists can relatively freely choose their styles or patterns in a *kolam* artwork, at the population-level, artists prefer and are biased toward specific (sequences of) gestures and prefer to remain in orthogonal gesture space. While a general tendency for specific gestures and geometric spaces exists on the population-level, artists can still widely differ in their stylistic variations or patterns in *kolam* art. In fact, the variation in styles and patterns in *kolam* art is dominated by artist-level variation and expertise. Thus, styles and patterns in *kolam* art can be mapped onto artists and their expertise and presumably their preferences.

Group-level variation can emerge naturally and become embedded in art through strategic decisions to communicate group affiliations and even through an unconscious population-level process of iterative learning and performance (McElreath et al., 2003). In contrast to predictions from ethnic marker theory that ethnic markers should be most prominent along cultural boundaries (McElreath et al., 2003), we show that *kolam* artworks only weakly covary along caste, neighborhood, or migration boundaries using a statistical approach. While the mapping between styles in *kolam* artworks and group affiliations is very limited, we still provide empirical evidence that there is scope for styles to become embedded in artwork as ethnic markers to signify group boundaries (for more details, see Bell et al., 2009). However, how much group-level variation is actually needed to function as an ethnic marker in a population is still unresolved.

Although art is a medium of communication and assorting with others with similar social norms or behaviors can facilitate successful coordination and cooperation, we believe that styles in art in this context do not play a coordinating role. One possible reason for the lack of covariation of artistic styles along cultural boundaries is that artists might have grown more similar to each other over time due to increased interactions (Healey et al., 2007; Granito et al., 2019). Due to the cross-sectional nature of our data, we might have not been able to see such a change over time. However, since the *kolam* tradition is considered community knowledge and artists share their designs with each other (Nagarajan, 2018), the development of styles might have been influenced by frequent intergroup contact, and shared attention and learning (Tomasello et al., 1993; Granito et al., 2019). Precisely, the notion of *kolam* art as community knowledge might have led to pressures to make artistic styles accessible and transparent to any potential audience.

The lack of migration history reflected in styles in *kolam* artwork could further indicate a pressure to conform to the dominant style in the region and the pressure to conform to the patrilocal style in the household. (Postmarital) Relocated *kolam* artists might adopt stylistic choices that are dominant in the region to communicate their belonging and similarity for successful group cooperation (Helbich and Dietler, 2008; Wallaert-Pêtre, 2008). Furthermore, the Tamil community in South India has a strongly patrilocal system of postmarital residence, thus relocated *kolam* artists might want to avoid communicating dissimilarity by adopting the local dominant style from their in-laws.

Avoiding conflicts with dissimilar others might be yet another reason why art, specifically styles in *kolam* art, have not developed to strongly map onto group boundaries and only show a limited scope for group coordination. Since individuals from different castes are often forced to cooperate with each other in the domain of farming in South India (Waring, 2012a), foreclosing valuable partnerships by using overt signals might not be desirable for successful cooperation (Smaldino, 2016). Art, specifically *kolam* art, might not be the preferred domain for ethnic markers to signal group membership since individuals have a multitude of (social) identities and group affiliations. Different situations and audiences might require different social identities that the artist can deliberately choose to occupy and display when suitable.

Instead, art might be a stage specifically reserved to promote artists' knowledge and expertise as well as their aesthetic preferences for symmetry, specific motifs, or gesture sequences through individual patterns and styles. Albeit artists may optimize their artistic displays toward a specific complexity “sweet spot” (Tran et al., 2021), our findings illustrate that they additionally strive to leave their individual mark on the artwork using their unique and distinct style for optimal distinctiveness (Brewer, 1991; Pickett et al., 2011). In fact, research in music has already shown that the probability of adopting a specific style disproportionately can be determined by frequency-based biases like conformity and novelty or prestige, success, and content biases (Brand et al., 2019; Youngblood, 2019). Since *kolam* art is culturally transmitted between artists, patterns or motifs might be subject to these transmission biases in social learning. For example, a novelty bias toward favoring unique patterns or motifs in *kolam* art could explain the substantial variation in kolam drawing styles between artists. Specifically, counter-dominance signaling could be a driving mechanism behind the cross-sectionally observed distinct styles between artists, since this mechanism posits that lower-status artists use highly unique styles to counter the currently dominating styles (Klimek et al., 2019). In order to investigate the cultural transmission of artistic traditions like *kolam* art and to disentangle the different biases at play, longitudinal data, explicit generative models, and careful consideration of the cost of adoption are required. Thus, our current data and analyses are insufficient to draw conclusions about cultural transmission processes and future research should explore this avenue in more detail.

How art can be mapped onto ethnic and cultural boundaries for the purpose of group coordination, and how much group-level variation is necessary to function as an ethnic marker are still vital questions to elucidate the coordinating role of art, and they require further investigations. Certainly, art and different aspects of art can actually play a crucial coordinating role depending on the context and the community. However, in Tamil Nadu, *kolam* designs might not be used as ethnic markers because there are already a variety of other ethnic markers that signify group membership. For instance, clothing, grooming, names, or the pottu (also known as bindi; see Davis, 1992, p. 25, for more information) can signify a woman's caste, religion, regional origin, class, and marital status (Mosse, 2018; Nagarajan, 2018). Furthermore, *kolam* designs might not be preferred as visual ethnic markers because their primary audiences are neighbors and visitors who most likely already know each other. This factor could explain a limited utility of *kolam* artwork serving as ethnic markers and further explain the lack of group mappings onto styles in *kolam* art. Another important consideration would be whether the groups are sufficiently distant to entail information about ethnic and cultural boundaries in the present sample. While we collected data from three different neighborhoods, the maximum distance between households was only slightly above 3 km. Additionally, caste endogamy persists, and thus women often still stay in the same caste community after postmarital relocation, even though artists in our sample migrated from different villages or cities of the states Karnataka, Kerala, and Tamil Nadu to Kodaikanal. Furthermore, although our sample consists of a mix of Scheduled Castes, Backward Castes, and Forward Castes, some caste groups within each of these categories are closely associated with each other or branches of the same caste community. Thus, our research only serves as an example of a systematic investigation of how styles in art can be mapped onto ethnic and cultural boundaries for group coordination by decomposing sequential behavior into states in a Markov chain. We demonstrate how describing a cultural system as a Markov chain can help us partition variation in styles to gain new evolutionary insights on the role of art for social coordination.

Certainly, loop patterns and motifs in *kolam* art are not limited to a sequential description using Markov chains, since ethnomathematicians have been intrigued by the many mathematical properties in *kolam* art (Layard, 1937; Ascher, 2002; Nagata, 2015). *Kolam* loop patterns are often structured by bilateral symmetry (i.e., vertical or horizontal symmetric) and rotational symmetry (see *kolam* drawings in Figures 2, 3). Furthermore, some complex *kolam* designs even exhibit fractal scaling by continuously repeating specific patterns. Symmetry, fractal scaling, geometric complexity, canvas size as well as the density of a *kolam* are all appreciated aesthetic qualities by Tamil women (Nagarajan, 2018, p. 165–167, 189), but not considered in the current analyses using Markov models. While the relation between complexity, density, and canvas size to individual and group-level variation has already been investigated (Tran et al., 2021), the relation between symmetrical and fractal properties of *kolam* designs to individual and group-level variation is still elusive and requires future explorations.

Future research should focus more on a synthesis between theoretical and statistical models and ethnographic methods in the field. For instance, classification tasks can be applied to motifs in art to gain a better understanding of the (cultural-historical) significance of specific motifs in art for social coordination (Bell, 2020). Bell (2020) demonstrated how triad classification tasks could be used to measure the signaling value of specifically selected Tongan motifs that are not constrained by functional sufficiency and thus elucidate their role for social coordination and the implied signaling dynamics. Similarly, specific *kolam* motifs with specific meaning, such as the “temple lamp” (see Figure 2A; Waring, 2012b) or auspicious symbols like the swastika (see Figure 1, left; Thomas, 1880) could be selected to measure their signaling value using the triad classification tasks. Our statistical approach and the methods proposed by Bell and Paegle (2021) could complement each other in future investigations of the coordinating roles of other art forms and material culture products. Another promising future avenue would be to expand ethnic marker theory and fieldwork to explain the dynamics between costly investments and ethnic markers. Since complex *kolam* designs require years of learning and practice, they can be considered costly. Similarly, Polynesian tattoos are markers of identity and political status, but costly due to their permanent and painful nature (Gell, 1993; Schildkrout, 2004). In these ritual examples, the evolutionary dynamics and trajectory of ethnic markers could deviate from current predictions of ethnic marker theory, and thus future research needs to consider costly ethnic markers.

In archaeology and evolutionary anthropology, a substantial amount of research has been dedicated to studying the step-by-step production of lithics (e.g., stone tools) and pottery (chaîne opératoire; Sellet, 1993). Our state-based Markov analysis could be extended and applied to investigate the technical processes and operational sequences involved in the production of material culture. Stylistic variations in *kolam* art and other artistic traditions can transcend specific motifs and patterns, and manifest themselves in methodological and technical processes of the art production. For instance, *kolam* artists can systematically differ in their use of rice powder or chalk or their sequence of loop additions with the potential aid of applying scaffolding techniques. According to Gosselain (1999), different technological stages in pottery production can be associated with different stylistic displays of groups of Bafia potters in Cameroon. Thus, different sequences or stages of the production can further reflect stylistic expressions along ethnic or cultural boundaries due to shared learning. In the context of art, methodological and technological differences, such as brushstroke, pigment or contouring sequences and technique could be investigated by describing the different behavioral sequences as states in a Markov system to elucidate whether stylistic variations follow cultural and ethnic boundaries. We believe that the potential application of our state-based Markov approach to material culture and art are vast and could lead to compelling new insights into the evolutionary importance and coordinating role of art and other cultural artifacts.

## 5. Conclusion

Using a state-based Markov approach to systematically study the link between styles or patterns in art and group affiliations, our findings inform discussions on the evolutionary role of art for group coordination by encouraging researchers to use systematic methods to measure the mapping between specific objects or styles onto groups.

We show on a Tamil artistic tradition case study that although art can be a medium of communication, it is not necessarily dominated by group affiliation. While distinctive styles or patterns of artistic design are not apparently linked to caste boundaries, neighborhoods, or previous migration, they are linked to artist-level variation, such as expertise and presumably preferences. Our findings corroborate our understanding that artistic traditions and behavior in this context are primarily a domain where individuals carve out a unique niche for themselves to differentiate themselves from others.

## Data Availability Statement

The *kolam* data and code for this study are available and can be found on GitHub: http://github.com/nhtran93/kolam_coordination. The R package to analyze kolam drawings can be further found on GitHub: http://github.com/nhtran93/kolam.

## Ethics Statement

The studies involving human participants were reviewed and approved by the Max Planck Institute for Evolutionary Anthropology and the University of California, Davis. Written informed consent to participate in this study was provided by the participants' legal guardian/next of kin.

## Author Contributions

N-HT and ŠK designed the analysis. N-HT, TMW, and BAB wrote data processing software. N-HT wrote the statistical software, conducted the analysis, and wrote the manuscript. ŠK reviewed the code. TMW collected the data and designed the *kolam* lexicon. SA led the data transcription team. BAB and TMW provided critical feedback. All authors provided edits and revisions.

## Funding

This work was supported by US NSF Doctoral Dissertation Research Improvement Grant #0823416 and the Max Planck Institute for Evolutionary Anthropology.

## Conflict of Interest

The authors declare that the research was conducted in the absence of any commercial or financial relationships that could be construed as a potential conflict of interest.

## Publisher's Note

All claims expressed in this article are solely those of the authors and do not necessarily represent those of their affiliated organizations, or those of the publisher, the editors and the reviewers. Any product that may be evaluated in this article, or claim that may be made by its manufacturer, is not guaranteed or endorsed by the publisher.

## Footnotes

^1^Nagarajan (2018, p. 53) describes *kolam*-making in the following way: “While the woman is creating it [kolam], she exhibits a set of gestures as a visual art form—a sequence of bodily movements requiring a quality of attentiveness akin to dance or yoga.”

^2^Note: For the purpose of our analysis, we neglected the three special, single gestures: c1, c2 and c3.

^3^Pseudo-Bayesian model averaging computes the relative model weights using Bayesian bootstrap (For more details, see the manual, Vehtari et al., 2020).

^4^For more details see icc and variance_decomposition functions in performance R package (Ludecke et al., 2021).

^5^Note that we have four transition matrices, so we computed the ICC separately according to the above described procedure for each of the transition matrices: across geometric space, orthogonal space, diagonal space and transitional space.
