# Supplementary material for: Limited Scope for Group Coordination in Stylistic Variations of Kolam Art
Source: Front Psychol. 2021 Oct 28;12:742577. doi: 10.3389/fpsyg.2021.742577 (PMC8584996; doi:10.3389/fpsyg.2021.742577)
Supplement: Supplementary file 1 [file Data_Sheet_1.pdf]

Supplementary Material

1 *KOLAM* DATA

1.0.1 Background Information and Description of Sample and Variables

Between 2007 and 2009, TMW lived in Kodaikanal, Tamil Nadu in India and investigated *kolam* drawings to study human cultural evolution in an artistic domain. This included the development of an expanded gestural lexicon to describe *kolam* drawings and an interactive software for transcribing *kolam* drawings (Waring, 2012). In spring 2009, TMW and local research assistants collected data according to a snowball sampling procedure on *kolam* drawings from mainly three neighborhoods of Kodaikanal, Tamil Nadu: Anna Nagar, Naidupuram and Munjikal. Kodaikanal is a municipality city in district of Dindigul, Tamil Nadu. According to Census India (2011), Kodaikanal with an area of 21.45 km^2 has a population of 36,501. 48.84% of the population are Hindus, 12.00% are Muslims, 38.69% are Christians and the remaining under 1% follow other religions.

The data set includes interview surveys, which contained demographic details on *kolam*-making practice, questions about *kolam* learning, and spatial location of artists' homes (GPS). A GPS tracker of type Garmin GPSmap 60 CSx was used. Furthermore, a structured sample of *kolam* drawings using pens in notebooks was collected. The survey further contains information on who artists learn from and who they were helping to learn or have taught *kolam*-making. TMW and the local research assistants asked women to share their personal and purchased practice notebooks.

Artists in our data set self-identify with a total of 19 different caste categories. These caste categories are associated with varying privilege and include local and migrant caste groupings. There were on average 10 women in a caste group. Scheduled Castes include Aathi Dravidar, Dhobi, Pariyar, Pallar and Asariyar. Chettiyar, Koundar, Mannadiyar, Mudaliyar, Naidu and Brahmin are Forward Castes. Nambothiri and Iyar are branches of the Brahmin community. All other castes are Backward Castes. We constructed 8 neighborhood clusters with on average 24 women in a neighborhood cluster. Further details on the samples and variables can be found in Tables S1 and S2.

Table S1. Descriptive statistics of the sample and variables used in the models.

	Mean	SD	Median	Min	Max	Levels
Age	31.88	10.08	30	15	60	
Duration of Practice	19.46	10.73	18	1	52	
Caste	-	-	-	-	-	19
Nativity	34 Yes, 158 No					2
Residence Distance (in metres)	864.72	686.63	618.42	0	3005.82	

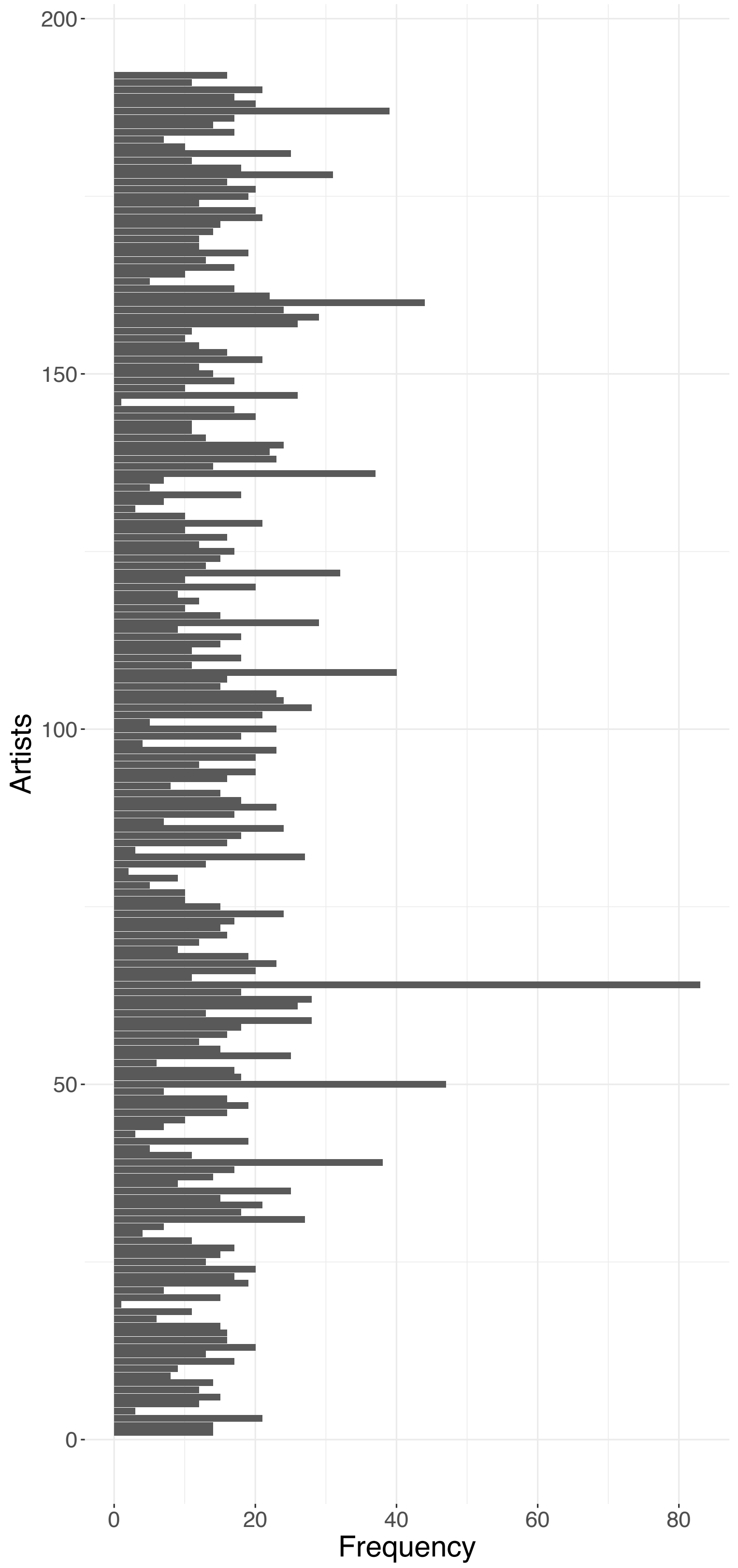

Figure S1. The frequency of *kolam* drawings by artists. Each bar on the y-axis represents one artist. The x-axis shows the number of *kolam* drawings by an artist. Median = 16 and Mean = 16.3 *kolam* drawings per woman.

Table S2. Caste

Caste	Artists
Aathi Dravidar	8
Asariyar	2
Ayyaraka	1
Brahmin	3
Chettiyar	2
Dhobi	39
Iyar	3
Koundar	1
Mannadiyar	2
Mudaliyar	24
Nadar	21
Naidu	34
Naiyakar	4
Nambothiri	1
Pallar	26
Pariyar	1
Thevar	11
Vanniyar	4
Vellalar	5

1.0.2 *Kolam* drawings and the lexicon of gestures

The square *kolam* drawings that we are focusing on have the advantage that we can map the drawings on a small identifiable set of gestures suitable for analyses. For this purpose, a lexicon of gestures was developed (Waring, 2012). We transcribed the *kolam* drawings using the lexicon and subsequently transferred them into a database using the *kolam* R package (Tran et al., 2020).

The gestures that constitute a loop and the *kolam* drawings can be categorized into three different geometric spaces with distinct characteristics: orthogonal, diagonal, and transitional (each set of gestures is represented by O, D, T, respectively). Figure S2 illustrates the three different geometric spaces in distinct colors and all the theoretically possible transitions between gestures and geometric categories. Each of these categories contains four gestures. The orthogonal gestures further contain two special variations (H and P). Every gesture is accessible from itself. Gestures from the same geometric category are transient and fully connected; thus, gestures can transition from or to other gestures of the same geometric category as well as remain in the same gesture (i.e., repeat the same gesture again). Transitions between gestures from different geometric categories are constrained: Diagonal gestures cannot transition to orthogonal gestures and vice versa. Orthogonal and diagonal gestures are only connected to each other via transitional gestures. Thus, transitional gestures can not only transition within their category, but can transition between categories to or from orthogonal and diagonal gestures. Self-loops are possible on the gesture level and the geometric space level. Furthermore, there are three special, decorative gestures that are merely single, stand-alone gestures and cannot be connected to any other gesture.

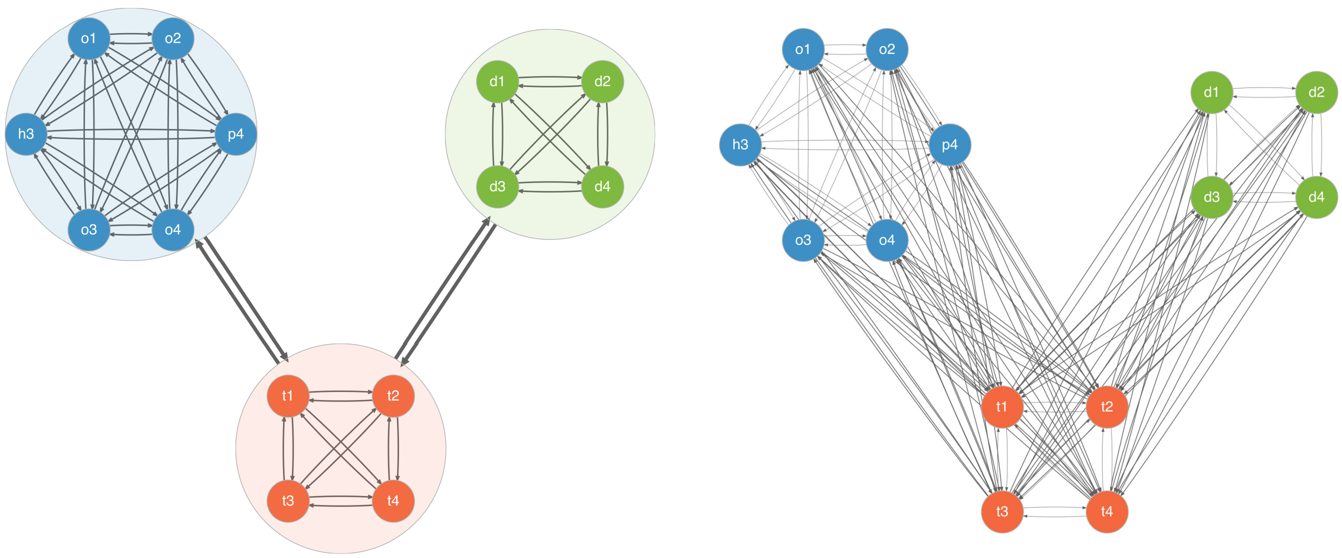

Figure S2. Transitions between orthogonal, diagonal and transitional gestures. Both transition networks depict 14 nodes corresponding to the 14 gestures and three clusters that correspond to the three geometric spaces: orthogonal (blue cluster), diagonal (green cluster), and transitional (orange cluster) spaces. The left transition network emphasizes the conditional probability of switching from a current geometric space to a different one (e.g., transitioning from orthogonal to transitional space) and illustrates that there are no direct transitions between orthogonal and diagonal gestures. The right transition network shows all the possible transitions gesture by gesture. The probability of remaining in the same geometric space or repeatedly using the same gesture is not depicted by a self-loop; however, conceptually, self-loops are possible for all gestures and geometric spaces.

2 STATISTICAL MODELS

The transition count matrix y_{ijk} of size 14×14 representing the count of transitions from state (i.e., gesture) j to state (i.e., gesture) k for artist i was used in the statistical models 2 to 5. Four transition count matrices y_{ijk} were used in the statistical models 6 and 7 with a transition matrix for transitions between geometric spaces of size 3×3, a transition matrix for transitions within orthogonal space of size 4×4, a transition matrix for transitions within diagonal space of size 4×4 and a transition matrix for transitions within transitional space of size 4×4.

2.1 Model 1: Null Model

In the null model, we did not model the effect of duration of practice and assumed no variation between individuals and castes on the transition count matrices.

$$\begin{aligned} y_{ij} &\sim \text{Multinomial}(\pi_j) \\ \pi_j &\sim \text{Dirichlet}(\underbrace{1, ..., 1}_{14}) \end{aligned} \tag{S1}$$

2.2 Model 2: Fixed Individual Variation Model

In the fixed individual variation model, we only modeled the individual variation in the transitions from j to k, but the extent to which the individual variation influences the transition probabilities to k is fixed across the rows j of the transition matrix y_i as well as individuals.

$$
\begin{aligned}
y_{ij} &\sim \text{Multinomial}(\pi_{ij}) \\
\pi_{ij} &= \text{softmax}(\theta_{ij}) \\
\theta_{ijk} &= \mu_{jk} + \sigma \times z_{ijk} \\
\mu_{jk} &\sim \mathcal{N}(0,1) \\
\text{z}_{\text{ijk}} &\sim \mathcal{N}(0,1) \\
\sigma &\sim \text{Gamma}(2,5)
\end{aligned}
\tag{S2}
$$

2.3 Model 3: Full Individual Variation model

In the full individual variation model, we allowed the individual variation to vary across the rows j of the transition matrix y_i.

$$
\begin{aligned}
y_{ij} &\sim \text{Multinomial}(\pi_{ij}) \\
\pi_{ij} &= \text{softmax}(\theta_{ij}) \\
\theta_{ijk} &= \mu_{jk} + \sigma_j \times z_{ijk} \\
\mu_{jk} &\sim \mathcal{N}(0,1) \\
\text{z}_{ijk} &\sim \mathcal{N}(0,1) \\
\sigma_j &\sim \text{Gamma}(2,5)
\end{aligned}
\tag{S3}
$$

2.4 Model 4: Fixed Variation Model with predictors

The fixed variation model with predictors extends the previous model with duration of practice as a predictor and caste variation fixed across the rows j of the transition matrix y_i.

$$
\begin{aligned}
y_{ij} &\sim \text{Multinomial}(\pi_{ij}) \\
\pi_{ij} &= \text{softmax}(\theta_{ij}) \\
\theta_{ijk} &= \mu_{jk} + \beta_{jk} \times \text{duration}_i + \sigma_{\text{ind}} \times z_{ijk} + \sigma_{\text{caste}} \times z_{cjk} \\
\mu_{jk} &\sim \mathcal{N}(0,1) \\
\text{z}_{ijk} &\sim \mathcal{N}(0,1) \\
\text{z}_{cjk} &\sim \mathcal{N}(0,1) \\
\sigma_{\text{ind}} &\sim \text{Gamma}(2,5) \\
\sigma_{\text{caste}} &\sim \text{Gamma}(2,5) \\
\beta_{jk} &\sim \mathcal{N}(0,1)
\end{aligned}
\tag{S4}
$$

2.5 Model 5: Full Individual Variation Model with predictors

In the full variation model, we allowed the individual variation to vary across the rows j of the transition matrix y_i and extended the previous model with duration of practice as a predictor and caste variation further allowed to vary across the rows j of the transition matrix y_i.

$$
\begin{aligned}
y_{ij} &\sim \text{Multinomial}(\pi_{ij}) \\
\pi_{ij} &= \text{softmax}(\theta_{ij}) \\
\theta_{ijk} &= \mu_{jk} + \beta_{jk} \times \text{duration}_i + \sigma_{\text{ind}_j} \times z_{ijk} + \sigma_{\text{caste}_j} \times z_{cjk} \\
\mu_{jk} &\sim \mathcal{N}(0,1) \\
\text{z}_{ijk} &\sim \mathcal{N}(0,1) \\
\text{z}_{cjk} &\sim \mathcal{N}(0,1) \\
\sigma_{\text{ind}_j} &\sim \text{Gamma}(2,5) \\
\sigma_{\text{caste}_j} &\sim \text{Gamma}(2,5) \\
\beta_{jk} &\sim \mathcal{N}(0,1)
\end{aligned}
\tag{S5}
$$

2.6 Model 6: Conditional Transition Model with main predictors

For each of the four matrices $m = \{\text{geometric spaces, orthogonal, diagonal, transitional}\}$, the model includes predictor of duration, and includes individual variation that itself varies across the rows of the matrices, as well as the caste variation that also varies across the rows of the matrices.

$$
\begin{aligned}
y_{ij}^{\text{m}} &\sim \text{Multinomial}(\pi_{ij}^{\text{m}}) \\
\pi_{ij}^{\text{m}} &= \text{softmax}(\theta_{ij}^{\text{m}}) \\
\theta_{ijk}^{\text{m}} = \mu_{jk}^{\text{m}} + \beta_{jk}^{\text{m}} \times \text{duration}_i + \sigma_{\text{ind}_j}^{\text{m}} \times \text{z}_{\text{ind}_{ijk}}^{\text{m}} &+ \sigma_{\text{caste}_j}^{\text{m}} \times \text{z}_{\text{caste}_{cjk}}^{\text{m}} \\
\mu_{jk}^{\text{m}} &\sim \mathcal{N}(0,1) \\
\text{z}_{\text{ind}_{ijk}}^{\text{m}} &\sim \mathcal{N}(0,1) \\
\text{z}_{\text{caste}_{cjk}}^{\text{m}} &\sim \mathcal{N}(0,1) \\
\sigma_{\text{ind}_j}^{\text{m}} &\sim \text{Gamma}(2,5) \\
\sigma_{\text{caste}_j}^{\text{m}} &\sim \text{Gamma}(2,5) \\
\beta_{jk}^{\text{m}} &\sim \mathcal{N}(0,1)
\end{aligned}
\tag{S6}
$$

2.7 Model 7 - Full Model: Conditional Transition Model with all predictors

This is our full model and best-performing model according to leave-one-out cross validation presented in the main text.

For each of the four matrices $m = \{\text{geometric spaces, orthogonal, diagonal, transitional}\}$, the model includes predictors of duration and migration (i.e., nativity), and includes individual variation that itself varies across the rows of the matrices, as well as the caste and neighborhood variations that also vary across the rows of the matrices.

$$\begin{aligned}
y_{ij}^{\text{m}} &\sim \text{Multinomial}(\pi_{ij}^{\text{m}}) \\
\pi_{ij}^{\text{m}} &= \text{softmax}(\theta_{ij}^{\text{m}}) \\
\theta_{ijk}^{\text{m}} &= \mu_{jk}^{\text{m}} + \beta_{\text{duration}_{jk}}^{\text{m}} \times \text{duration}_i + \beta_{\text{native}_{jk}}^{\text{m}} \times \text{native}_i \\
&\quad + \sigma_{\text{ind}_j}^{\text{m}} \times \text{z}_{\text{ind}_{ijk}}^{\text{m}} + \sigma_{\text{caste}_j}^{\text{m}} \times \text{z}_{\text{caste}_{cjk}}^{\text{m}} + \sigma_{\text{residence}_j}^{\text{m}} \times \text{z}_{\text{residence}_{rjk}}^{\text{m}} \\
\mu_{jk}^{\text{m}} &\sim \mathcal{N}(0,1) \\
\text{z}_{\text{ind}_{ijk}}^{\text{m}} &\sim \mathcal{N}(0,1) \\
\text{z}_{\text{caste}_{cjk}}^{\text{m}} &\sim \mathcal{N}(0,1) \\
\text{z}_{\text{residence}_{rjk}}^{\text{m}} &\sim \mathcal{N}(0,1) \\
\sigma_{\text{ind}_j}^{\text{m}} &\sim \text{Gamma}(2,5) \\
\sigma_{\text{caste}_j}^{\text{m}} &\sim \text{Gamma}(2,5) \\
\sigma_{\text{residence}_j}^{\text{m}} &\sim \text{Gamma}(2,5) \\
\beta_{\text{duration}_{jk}}^{\text{m}} &\sim \mathcal{N}(0,1) \\
\beta_{\text{native}_{jk}}^{\text{m}} &\sim \mathcal{N}(0,1)
\end{aligned} \tag{S7}$$

3 MODEL COMPARISON

Table S3. Pseudo-Bayesian Model Averaging Weights with Bayesian bootstrap

	weights
Null Model	0.00
Fixed Individual Variation Model	0.00
Full Individual Variation Model	0.00
Fixed Variation Model with predictors	0.00
Full Individual Variation Model with predictors	0.00
Conditional Transition Model with main predictors	0.21
Full Model: Conditional Transition Model with all predictors	0.79

Table S4. Stacking weights of predictive distributions

	weights
Null Model	0.26
Fixed Individual Variation Model	0.01
Full Individual Variation Model	0.01
Fixed Variation Model with predictors	0.01
Full Individual Variation Model with predictors	0.00
Conditional Transition Model with main predictors	0.27
Full Model: Conditional Transition Model with all predictors	0.44

4 FULL MODEL (MODEL 7) RESULTS

4.1 Visual MCMC diagnostics

All $\widehat{R}$ values were less than 1.01, and visual inspection of trace plots, rank histograms and pairs plots indicated convergence of all models. We have added the trace plots of the estimated parameters for the transition matrix across geometric spaces to illustrate that our model converged. However, since our model estimates many parameters and multiple transition matrices, we refrain from plotting over 100 traceplots. Our open repository provides the data and code to fit all of the Bayesian transition models in order to reproduce our results and further contains code to produce trace, rank histogram and autocorrelation plots.

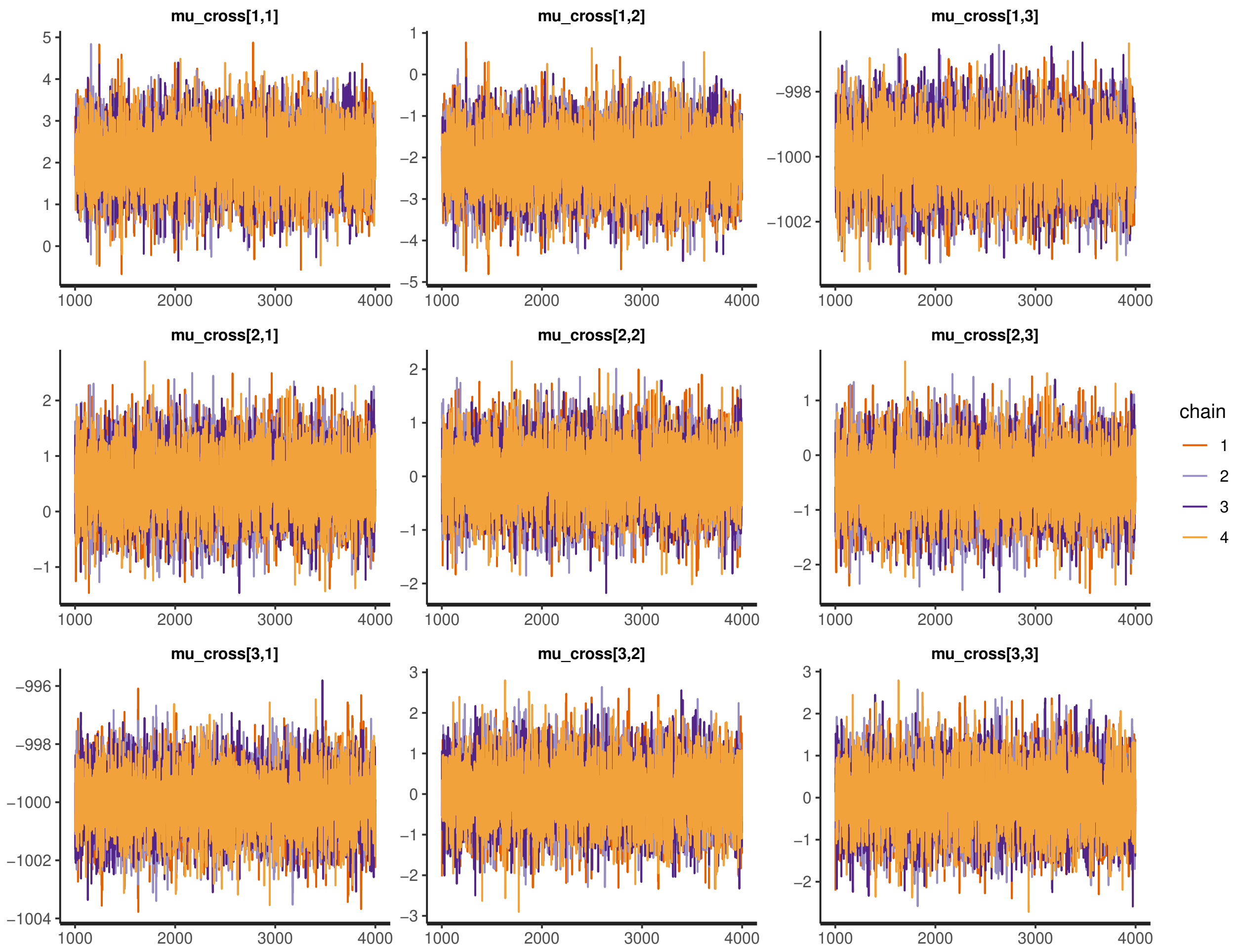

Figure S3. Traceplot of the population-level mean transition matrix of transitioning between geometric spaces showing mixing across chains and convergence.

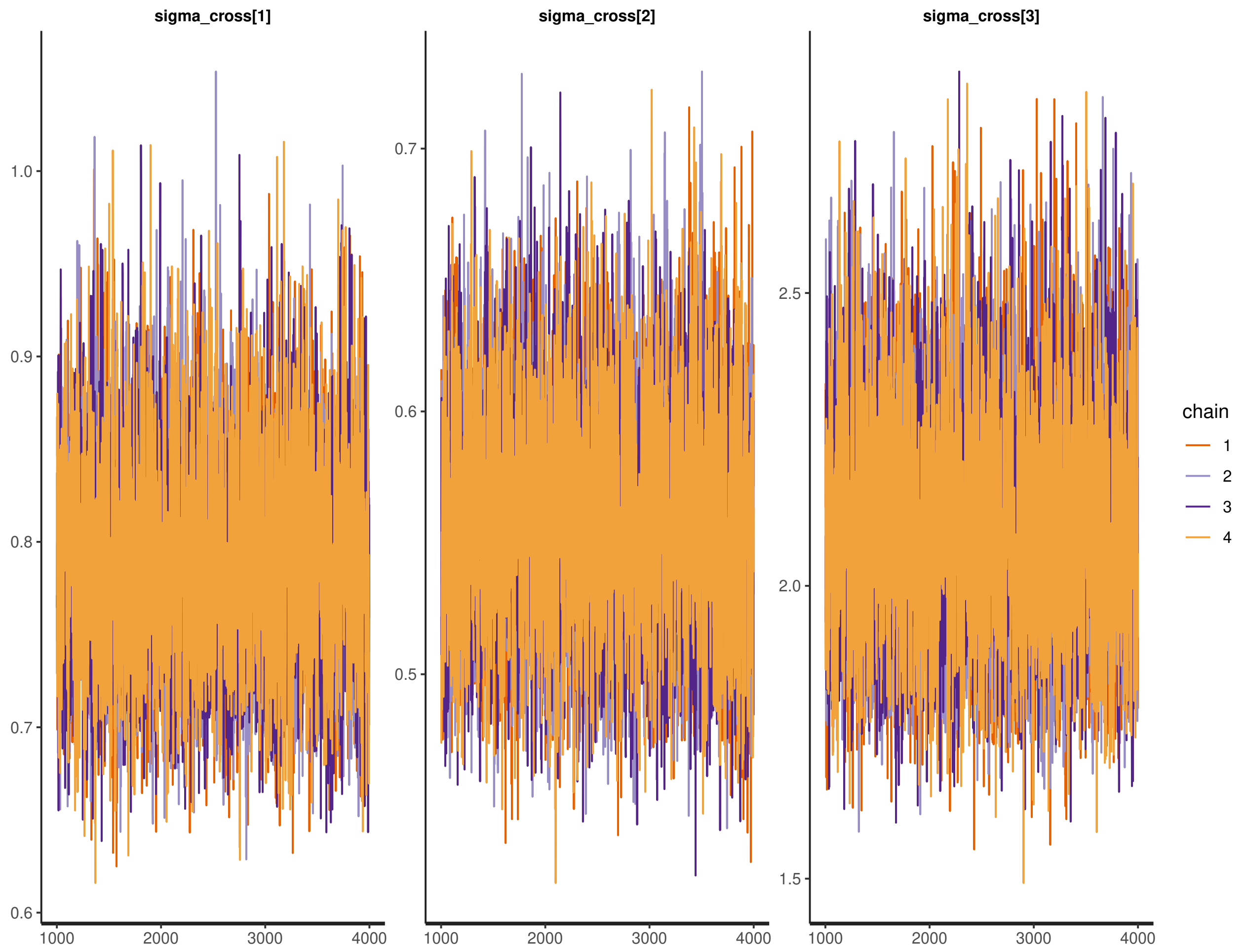

Figure S4. Traceplot of the individual variation in the transition matrix of transitioning between geometric spaces showing mixing across chains and convergence.

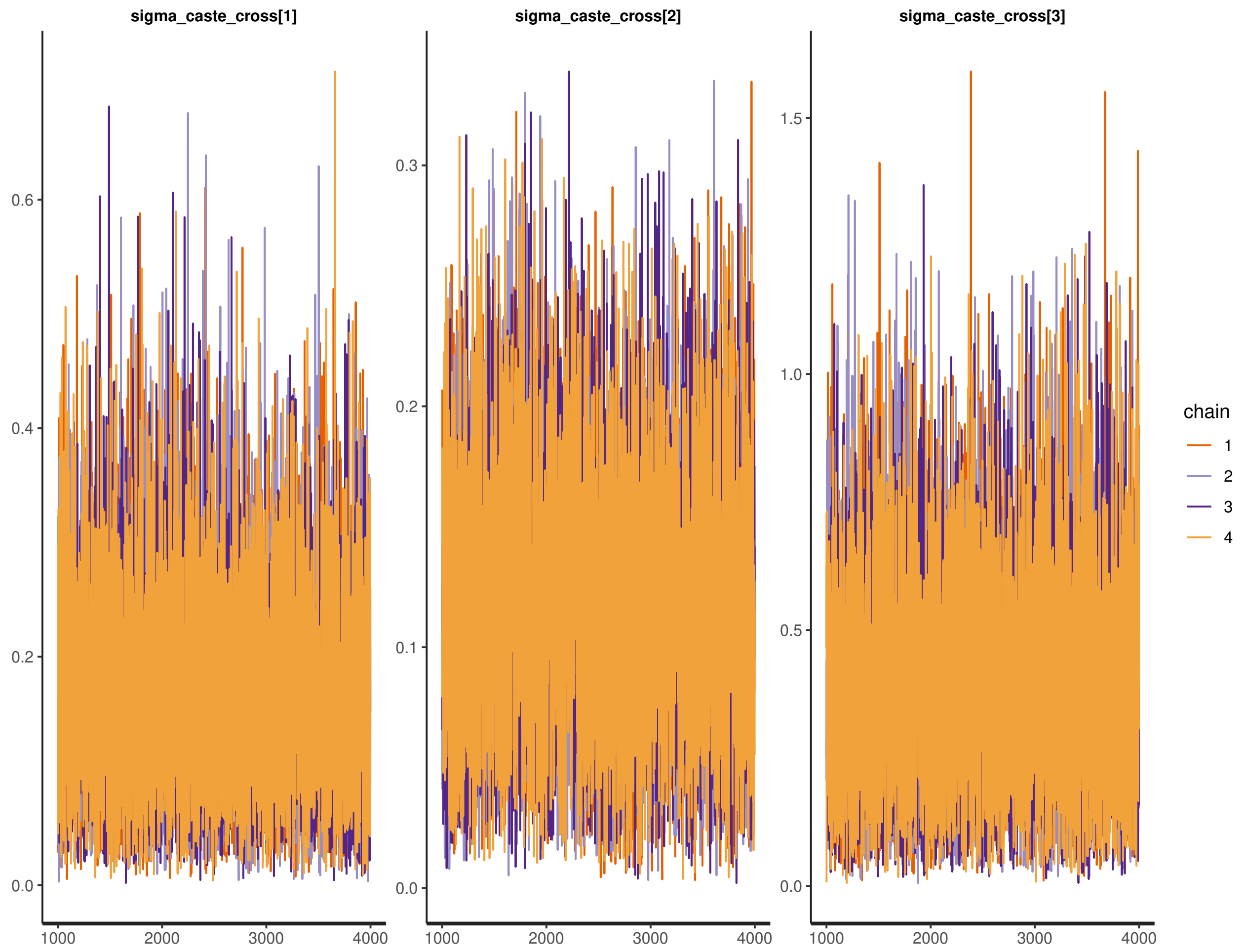

Figure S5. Traceplot of the caste variation in the transition matrix of transitioning between geometric spaces showing mixing across chains and convergence.

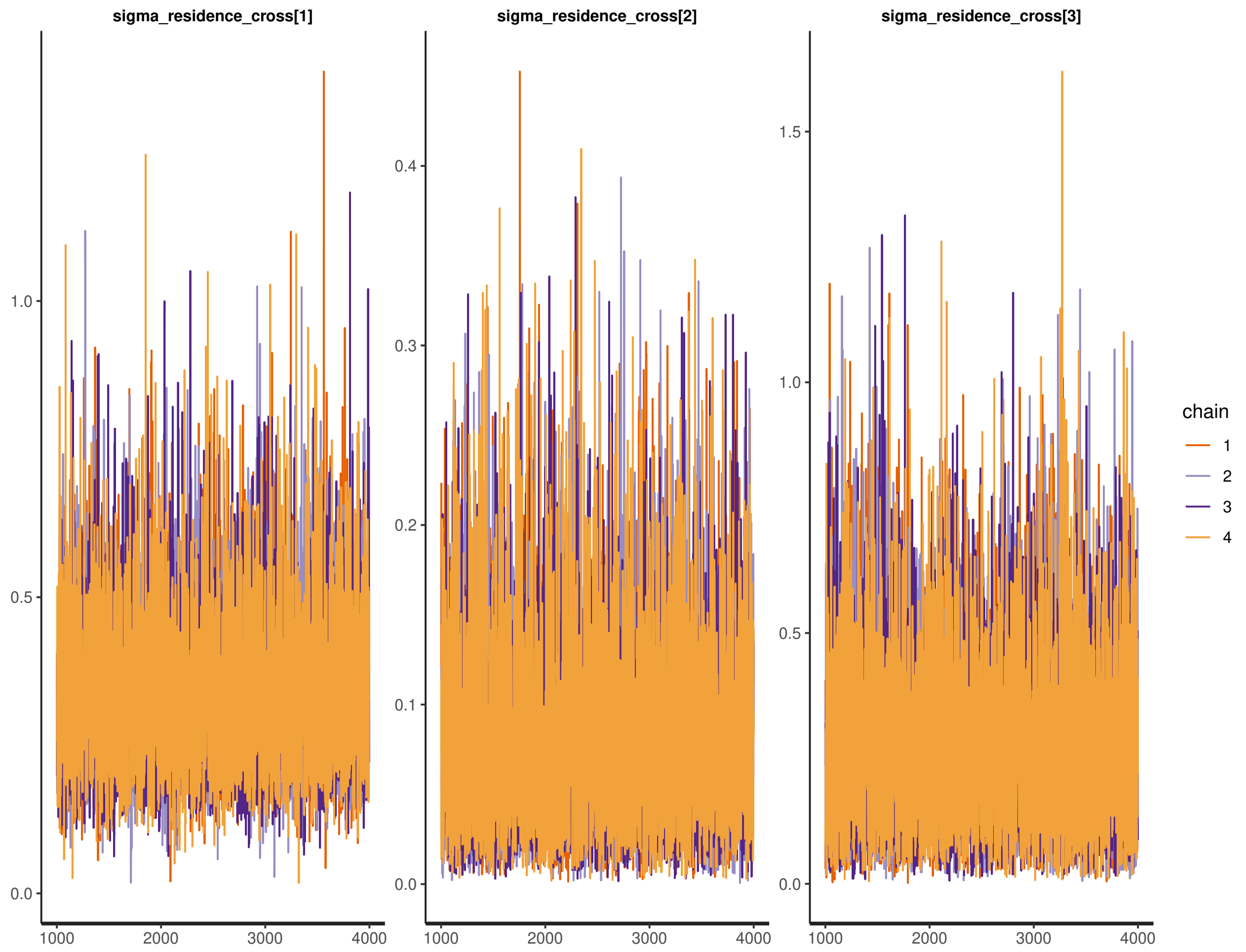

Figure S6. Traceplot of the neighborhood variation in the transition matrix of transitioning between geometric spaces showing mixing across chains and convergence.

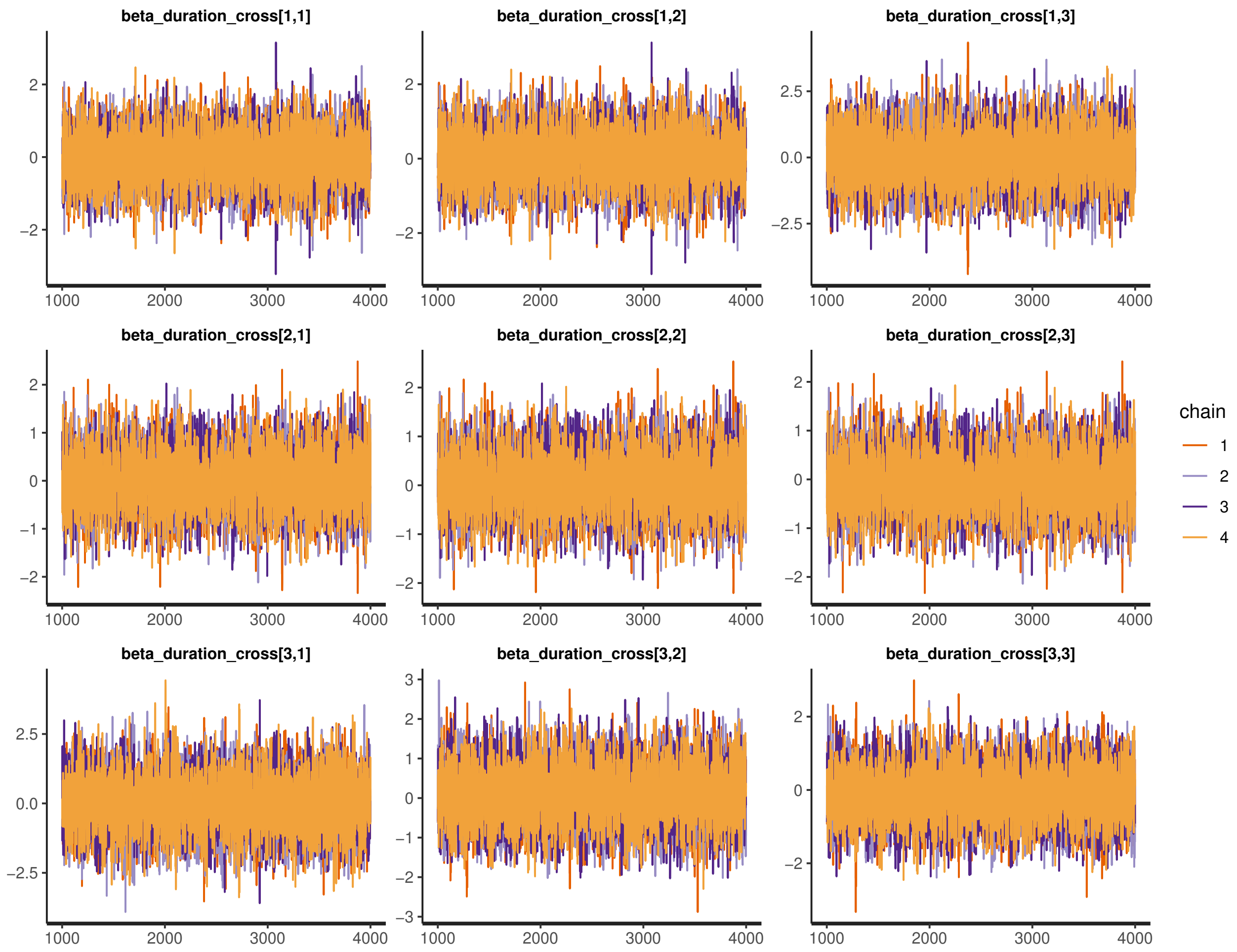

Figure S7. Traceplot of the effect of the duration of practice in the transition matrix of transitioning between geometric spaces showing mixing across chains and convergence.

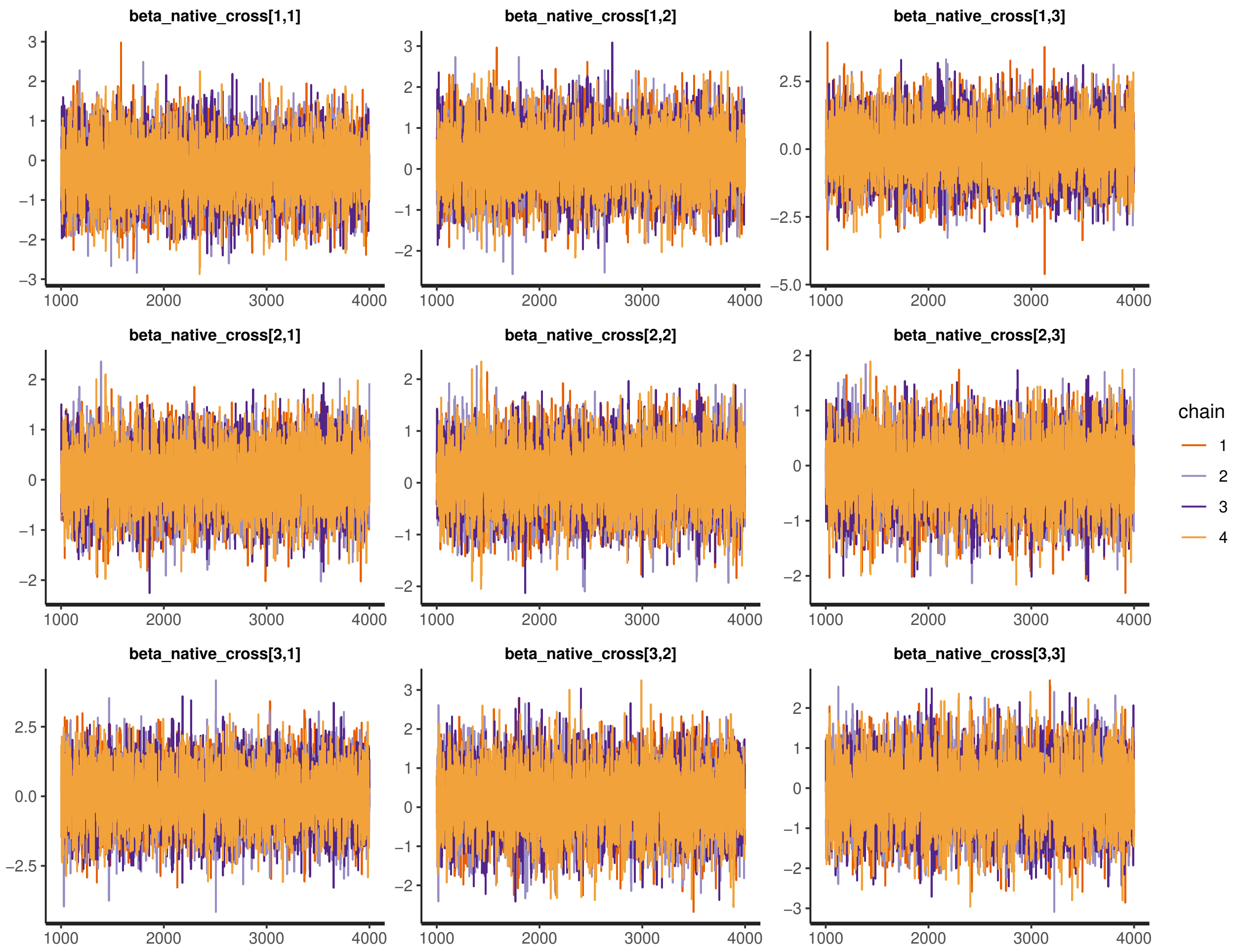

Figure S8. Traceplot of the effect of the migration history (nativity) in the transition matrix of transitioning between geometric spaces showing mixing across chains and convergence.

4.2 Population-level Results

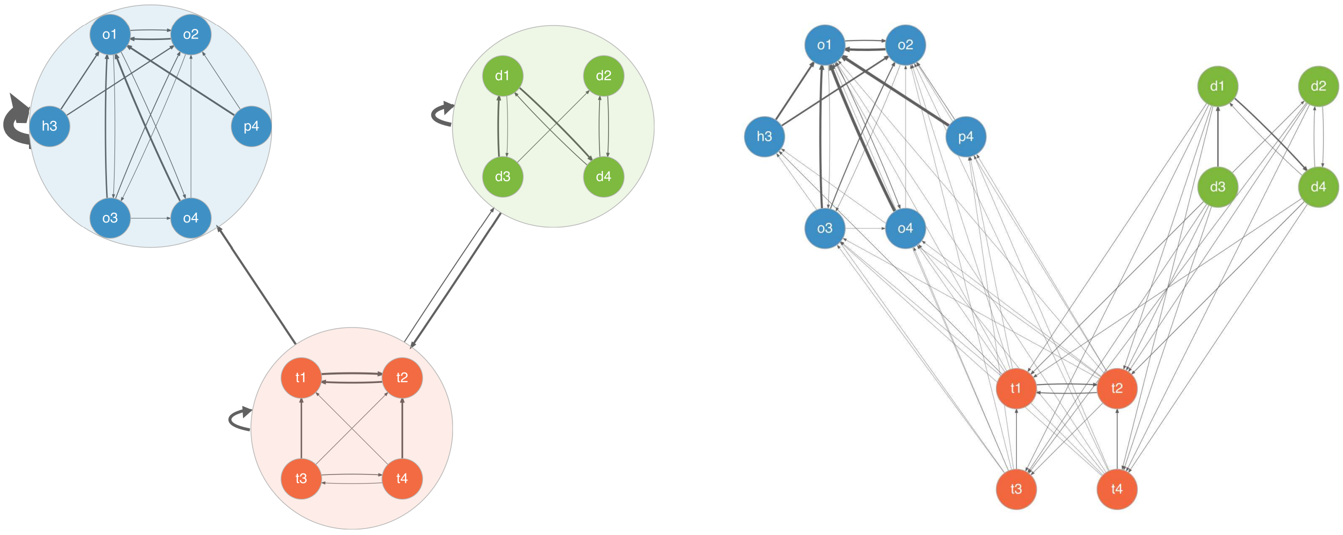

Figure S9. Estimated population-level transitions between gestures. The left state transition diagram emphasises the probability of transitioning between orthogonal, diagonal, and transitional gestures. The right state transition diagram illustrates all transitions between gestures within the same geometric space and between different geometric spaces. The width of the edges reflects the probability of transition, whereby a wider or bolder edge implies an increased probability of transition. The colours represent the three different geometric categories. The self-loops for each gesture are not depicted. Self-loops for the geometric categories on the right transition network are not depicted.

The ability to describe *kolam* art as a Markovian system further allows us to compute the stationary distribution after a sufficiently long time. The stationary distribution or the state distribution of the system at equilibrium describes the proportion of time that the Markov chain (sequence of gestures) is in any given state (i.e., gesture within a geometric space). The stationary distribution π can be found using the transition matrix T and by setting an initial distribution for π (π is a row vector of probabilities over the state space S), so that $\pi = \pi \mathrm{T}$.

On the population-level, our results illustrate that although artists are unconstrained in their patterns or stylistic variation in *kolam* drawings, and they can freely transition back and forth between geometric spaces, artists have evident preferences and biases towards certain gestures and geometric spaces. As seen in Figure S9, *kolam* patterns that arise in orthogonal geometric space are predicted to stay in orthogonal geometric space with a probability of 0.985 and transitioning to a different geometric space to access a greater diversity of gestures hardly occurs (probability of 0.015). Therefore, when an artist draws *kolam* patterns in orthogonal space, they are unlikely to transition between different geometric spaces and only draw patterns with different gestures within the orthogonal space. However, if artists draw *kolam* patterns in diagonal or transitional space, they tend to use a diverse set of gestures that span across multiple different geometric spaces. Considering that transitioning away from orthogonal space hardly occurs and concomitantly transitioning into orthogonal space occurs relatively frequently, the probability of orthogonal gestures at equilibrium is very high with 0.96. Thus, *kolam* artworks predominantly arise and remain in orthogonal space. Conversely, *kolam* patterns with gestures from transitional and diagonal space are very rare (0.03 and 0.01 respectively). Viewed at the population-level, our results show that transitions within geometric spaces are not equal, but there are evident biases for certain transitions. For instance, in

orthogonal space, there are no transitions between *h3* and *o4* or *p4* and *o3*, and in diagonal space there are no transitions between *d1* and *d2*.

4.3 Estimated Population-level Transition Matrices

Table S5. Estimated Posterior Transition Matrix within Orthogonal Space

	o1	o2	o3	o4	h3	p4
o1	0.41	0.30	0.10	0.18	0.01	0.00
o2	0.68	0.16	0.11	0.05	0.01	0.00
o3	0.61	0.28	0.03	0.08	0.00	0.00
o4	0.84	0.09	0.06	0.00	0.00	0.00
h3	0.53	0.46	0.00	0.01	0.00	0.00
p4	0.82	0.15	0.02	0.00	0.00	0.00

Table S6. Estimated Posterior Transition Matrix within Diagonal Space

	d1	d2	d3	d4
d1	0.08	0.05	0.09	0.78
d2	0.03	0.69	0.01	0.27
d3	0.76	0.15	0.05	0.04
d4	0.25	0.27	0.00	0.47

Table S7. Estimated Posterior Transition Matrix within Transitional Space

	t1	t2	t3	t4
t1	0.03	0.95	0.02	0.01
t2	0.81	0.18	0.00	0.01
t3	0.63	0.10	0.04	0.22
t4	0.08	0.76	0.11	0.05

4.4 Estimated Effects of Migration and Duration of Practice

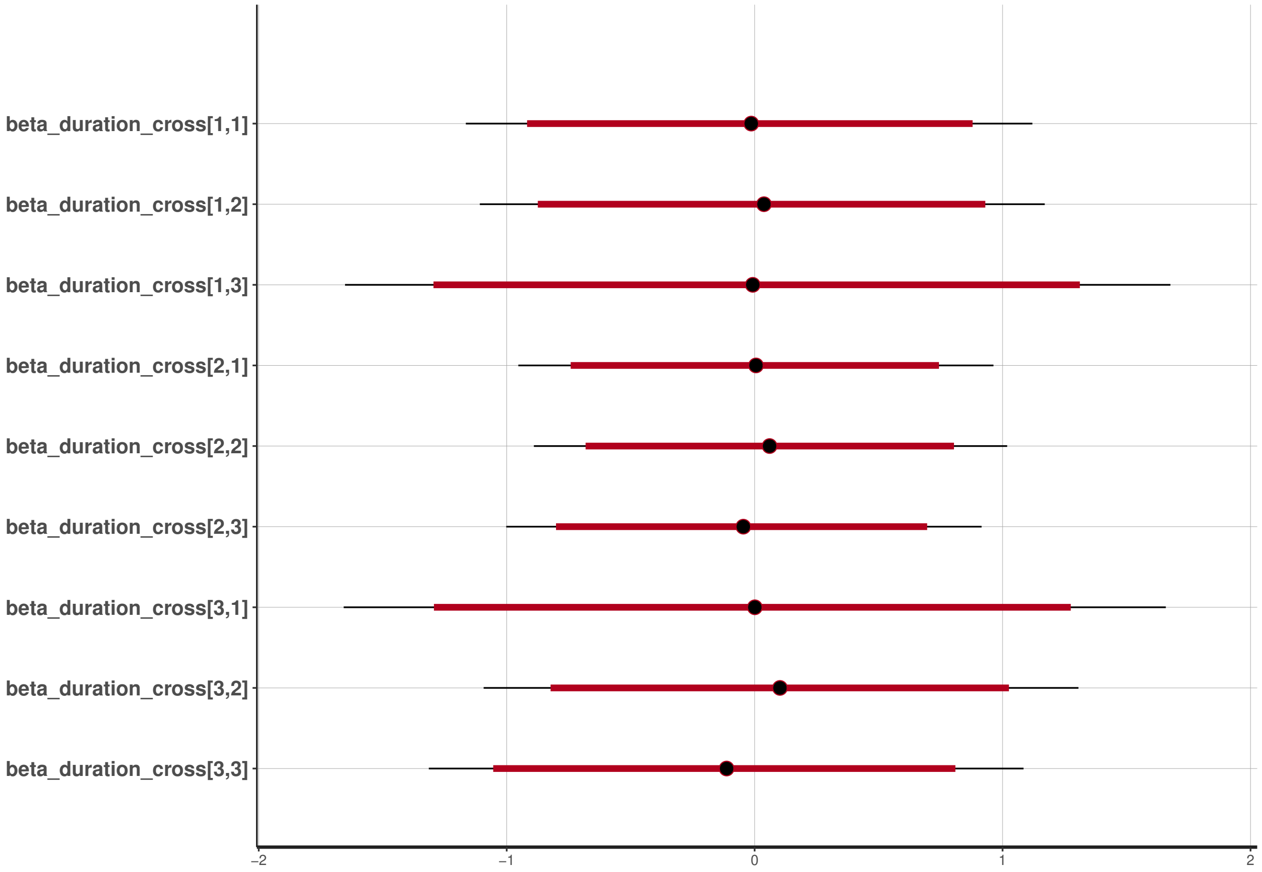

Figure S10. Estimated coefficients of the duration of practice on the transition matrix across geometric spaces. On the x-axis, the red interval shows the 90% HPDI interval and the black interval shows the 80% HPDI interval around the posterior mean.

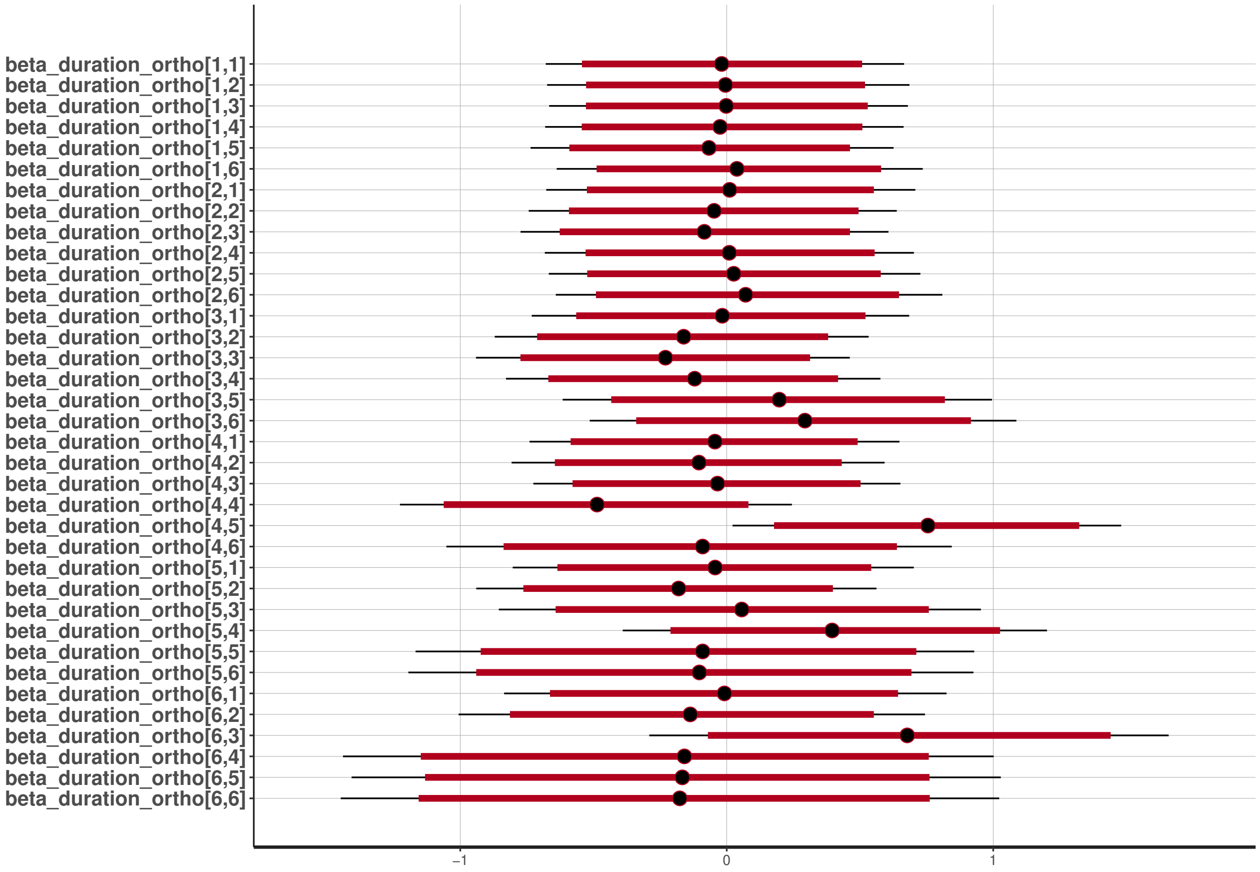

Figure S11. Estimated coefficients of the duration of practice on the transition matrix within orthogonal space. On the x-axis, the red interval shows the 90% HPDI interval and the black interval shows the 80% HPDI interval around the posterior mean.

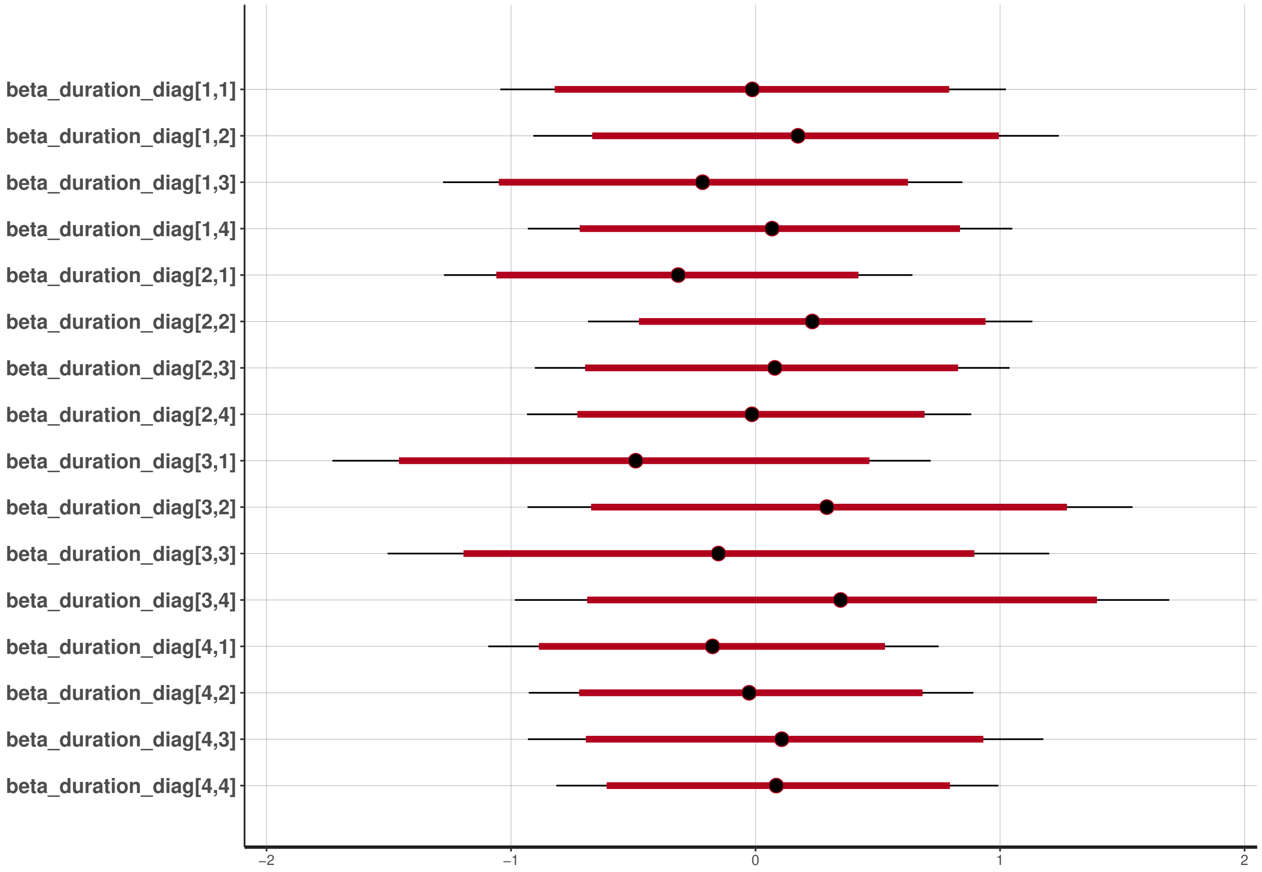

Figure S12. estimated coefficients of the duration of practice on the transition matrix within diagonal space. On the x-axis, the red interval shows the 90% HPDI interval and the black interval shows the 80% HPDI interval around the posterior mean.

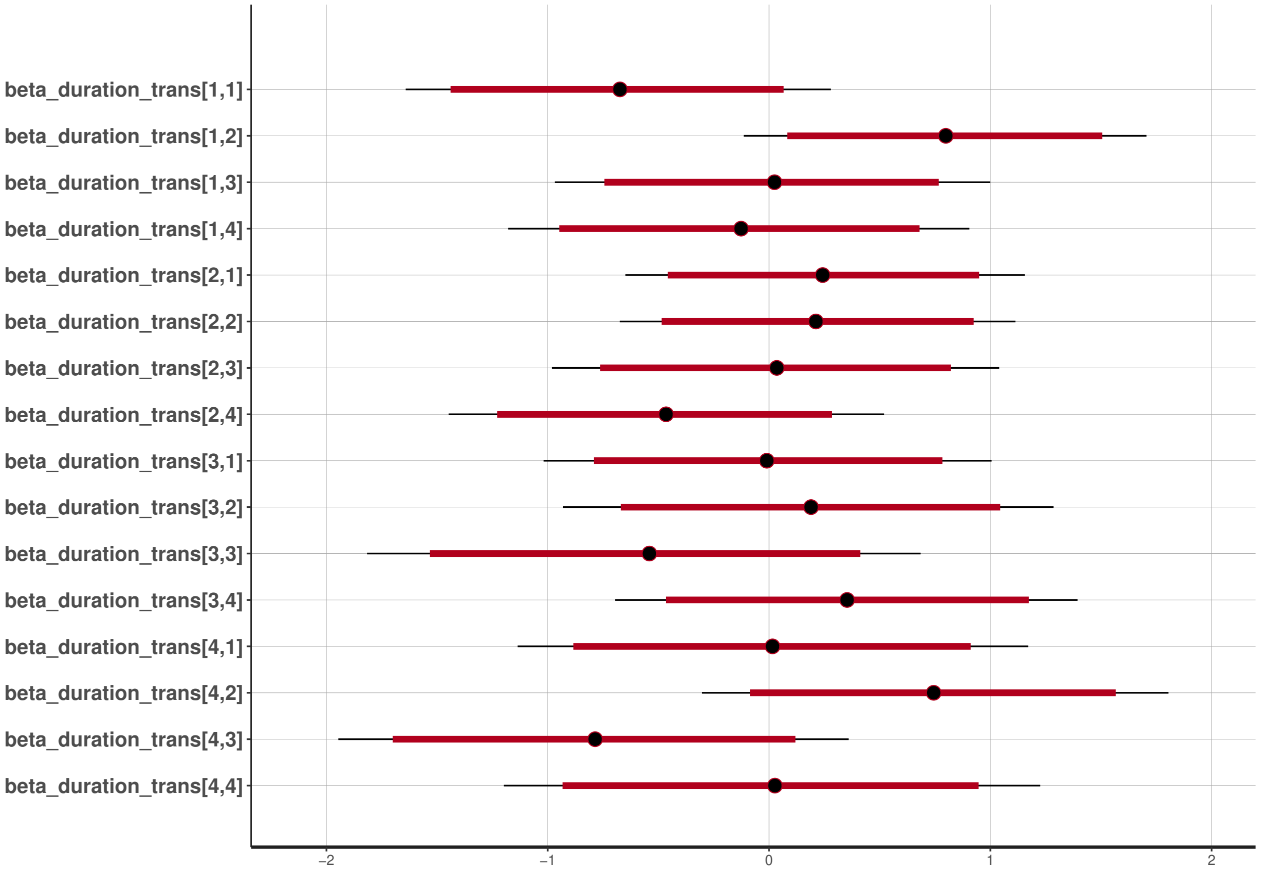

Figure S13. estimated coefficients of the duration of practice on the transition matrix within transitional space. On the x-axis, the red interval shows the 90% HPDI interval and the black interval shows the 80% HPDI interval around the posterior mean.

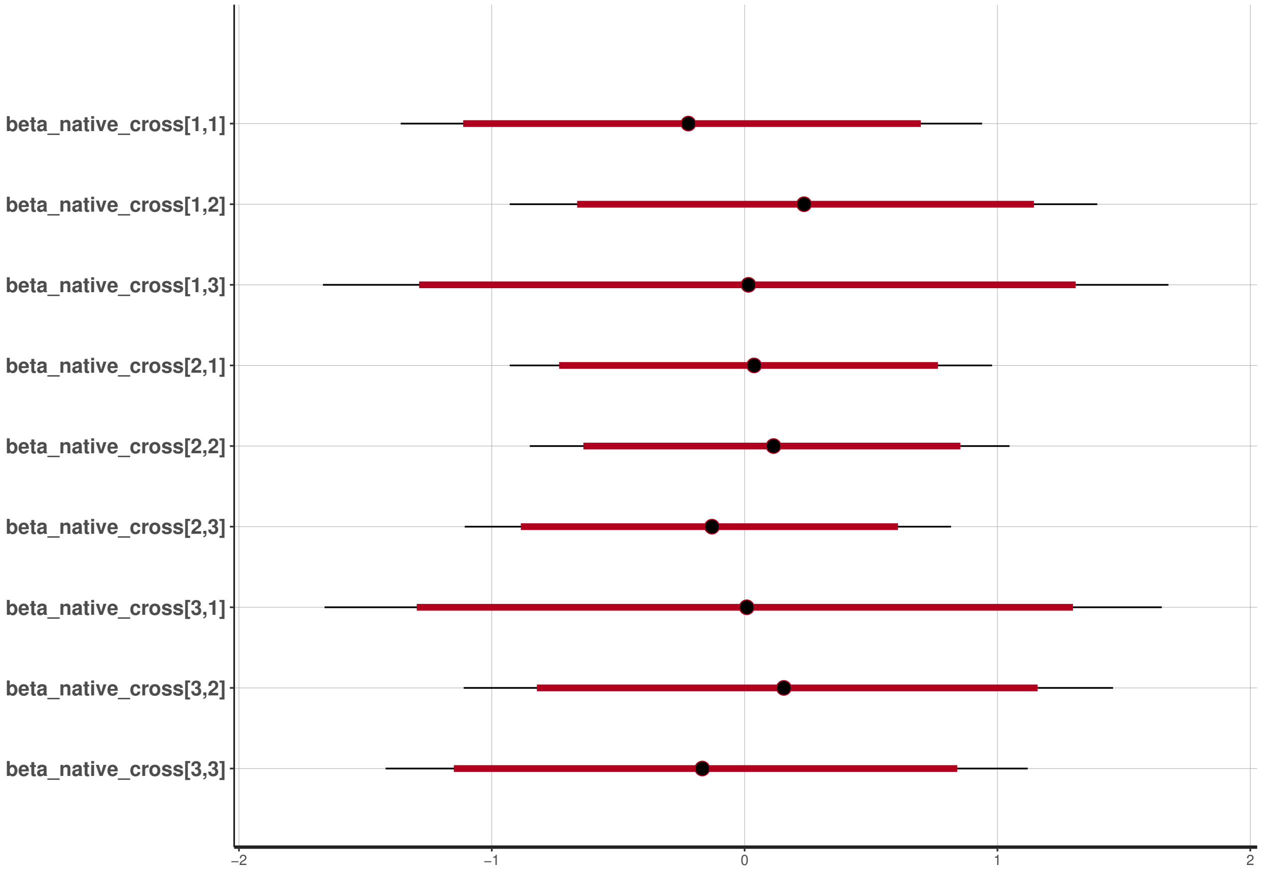

Figure S14. Estimated coefficients of the migration history (nativity) on the transition matrix across geometric spaces. On the x-axis, the red interval shows the 90% HPDI interval and the black interval shows the 80% HPDI interval around the posterior mean.

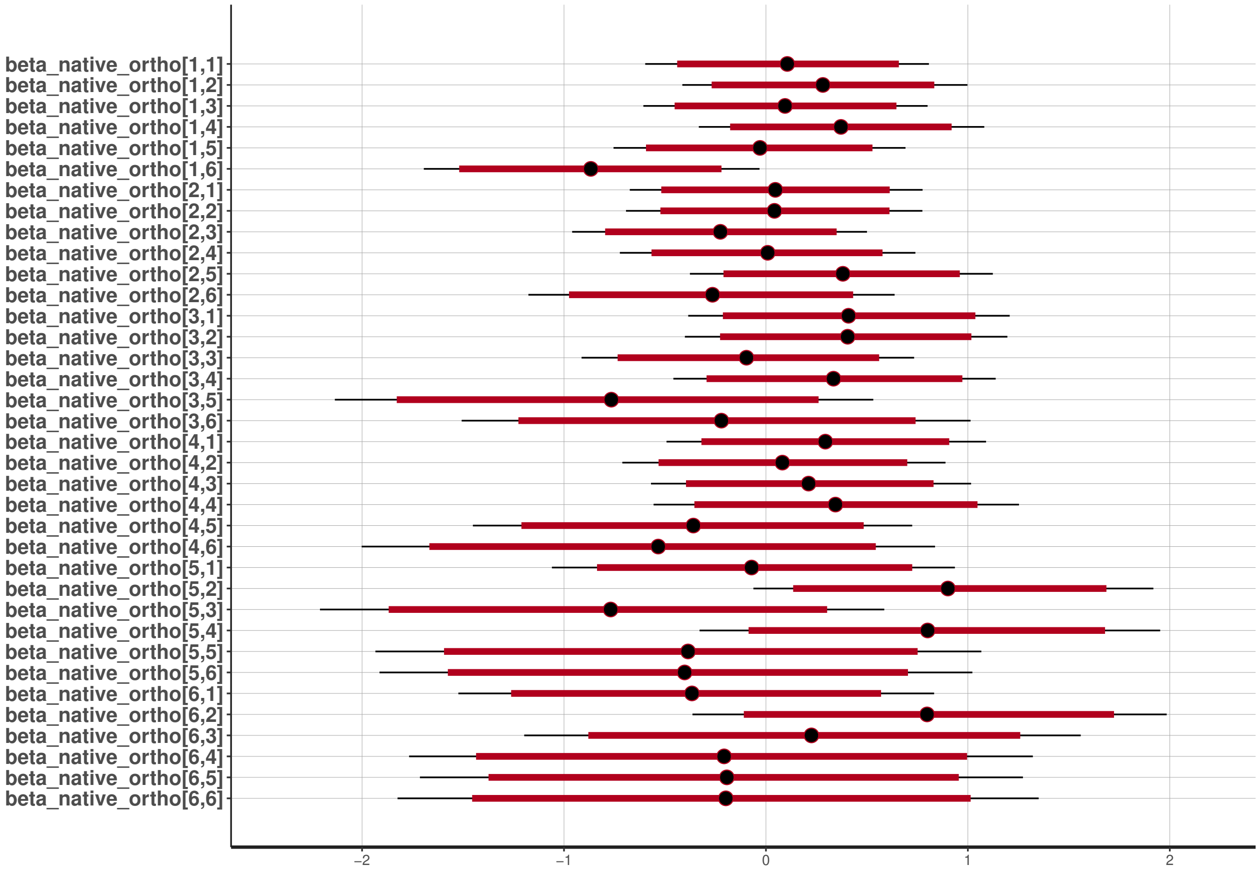

Figure S15. Estimated coefficients of the migration history (nativity) on the transition matrix within orthogonal space. On the x-axis, the red interval shows the 90% HPDI interval and the black interval shows the 80% HPDI interval around the posterior mean.

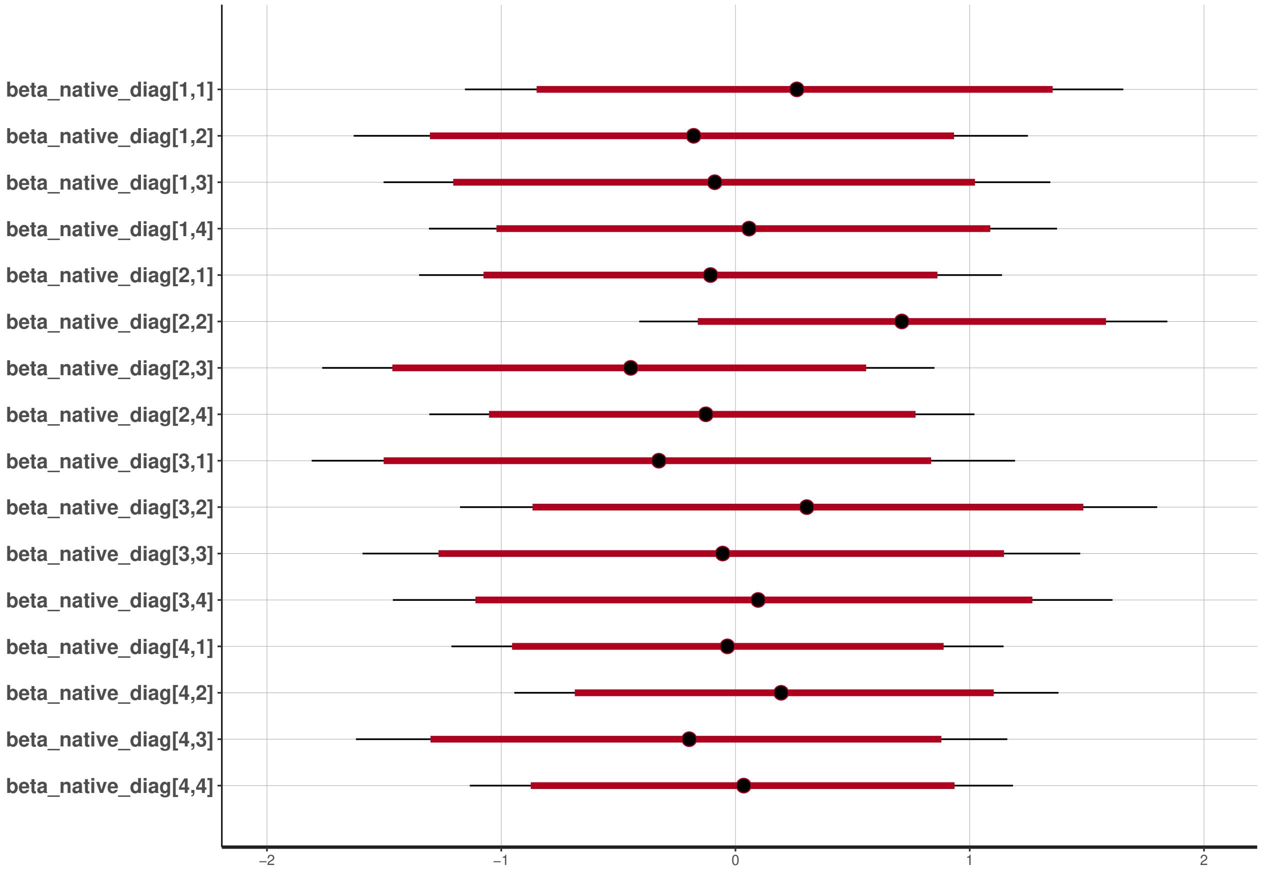

Figure S16. Estimated coefficients of the migration history (nativity) on the transition matrix within diagonal space. On the x-axis, the red interval shows the 90% HPDI interval and the black interval shows the 80% HPDI interval around the posterior mean.

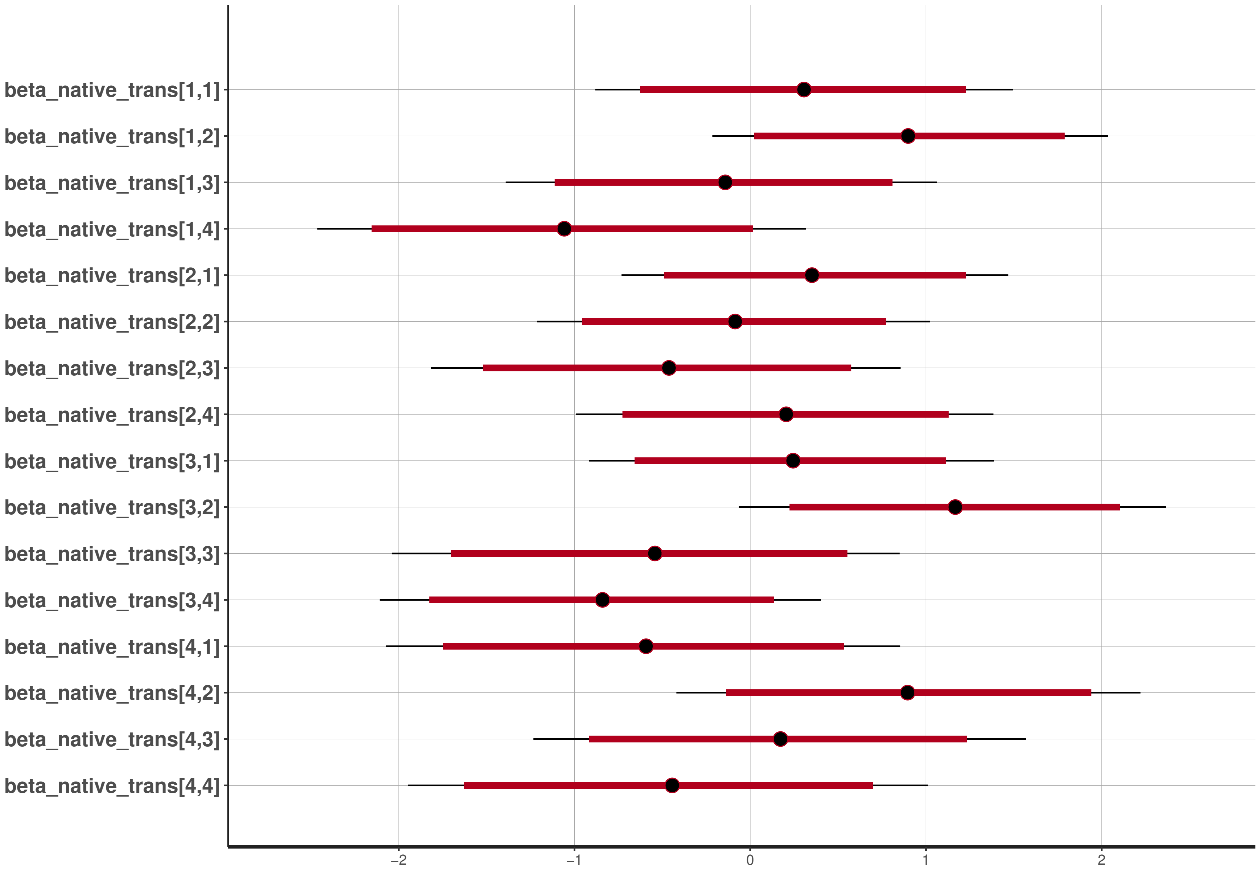

Figure S17. Estimated coefficients of the migration history (nativity) on the transition matrix within transitional space. On the x-axis, the red interval shows the 90% HPDI interval and the black interval shows the 80% HPDI interval around the posterior mean.

4.5 Equilibrium State Vectors

Over time Markov chains automatically find their equilibrium distribution. The equilibrium is the position where there is no more change in the distributions.

4.5.1 Population-level

Table S8. Equilibrium State Vector for the Between Geometric Space Transitions

orthogonal	transitional	diagonal
0.96	0.03	0.01

Table S9. Equilibrium State Vector for the Within Orthogonal Space Transitions

o1	o2	o3	o4	h3	p4
0.54	0.24	0.09	0.12	0.01	0.00

Table S10. Equilibrium State Vector for the Within Diagonal Space Transitions

d1	d2	d3	d4
0.15	0.41	0.02	0.42

Table S11. Equilibrium State Vector for the Within Transitional Space Transitions

t1	t2	t3	t4
0.45	0.53	0.01	0.01

4.5.2 Caste

Table S12. Equilibrium State Vector for the Between Geometric Space Transitions by Caste

Caste	orthogonal	transitional	diagonal
1	0.961	0.026	0.012
2	0.964	0.026	0.010
3	0.960	0.029	0.011
4	0.960	0.029	0.011
5	0.959	0.030	0.011
6	0.960	0.030	0.010
7	0.963	0.027	0.010
8	0.961	0.029	0.010
9	0.959	0.030	0.011
10	0.969	0.022	0.008
11	0.959	0.030	0.011
12	0.960	0.029	0.012
13	0.956	0.032	0.011
14	0.959	0.030	0.011
15	0.959	0.031	0.010
16	0.956	0.032	0.012
17	0.958	0.030	0.012
18	0.959	0.031	0.011
19	0.960	0.028	0.012

4.5.3 Neighborhood

Table S13. Equilibrium State Vector for the Between Geometric Space Transitions by Neighborhood

Neighborhood	orthogonal	transitional	diagonal
1	0.939	0.044	0.017
2	0.962	0.027	0.011
3	0.950	0.037	0.012
4	0.979	0.015	0.006
5	0.959	0.029	0.012
6	0.967	0.025	0.009
7	0.967	0.024	0.009
8	0.967	0.024	0.009

4.5.4 Native

Table S14. Equilibrium State Vector for the Between Geometric Space Transitions by Nativity

Nativity	orthogonal	transitional	diagonal
native	0.961	0.029	0.011
non-native	0.943	0.044	0.012

REFERENCES

[Dataset] Census India (2011). Kodaikanal Population Census 2011

[Dataset] Tran, N.-H., Waring, T., and Beheim, B. A. (2020). kolam R package

Waring, T. M. (2012). Sequential Encoding of Tamil Kolam Patterns. *Forma* 27, 83–92
